# The efferocytosis dilemma: how neutrophil extracellular traps and PI3K/Rac1 complicate diabetic wound healing

**DOI:** 10.1186/s12964-025-02092-4

**Published:** 2025-02-21

**Authors:** Yulin Xie, Jiaman Yang, He Zhu, Rongya Yang, Yunlong Fan

**Affiliations:** 1 Zhujiang Hospital, The Second School of Clinical Medicine, Southern Medical University, Southern Medical University, Guangzhou, 510599 China; 2 Department of Dermatology, The Seventh Medical Center of Chinese PLA General Hospital, Beijing, 100700 China; 3 Chinese PLA Medical School, Beijing, 100853 China

**Keywords:** NETs, Efferocytosis, Diabetes, Wound healing, Macrophage

## Abstract

**Aims/hypothesis:**

The resolution of apoptotic cells (ACs) is crucial for wound healing and tissue remodeling and is often impaired by persistent inflammation. This study aimed to elucidate the impact of neutrophil extracellular traps (NETs) on diabetic wound healing by targeting the phosphoinositide 3-kinase/Ras-related C3 botulinum toxin substrate 1 (PI3K/Rac1) signaling pathway, which is pivotal for macrophage efferocytosis.

**Methods:**

A streptozotocin-induced diabetic mouse model was used to assess the impact of NETs on efferocytosis in vivo. The effects of NETs on macrophage efferocytosis and wound healing were evaluated using specific inhibitors and agonists targeting the PI3K/Rac1 pathway. In vitro, macrophages from diabetic wounds or cell lines (Raw264.7) were treated with NETs and a panel of pharmacological agents of the PI3K/Rac1 pathway to evaluate macrophage efferocytosis.

**Results:**

NETs were found to inhibit macrophage efferocytosis, resulting in delayed clearance of ACs that accumulate within the wounds. Inhibition of NET formation in diabetic mice rescued impaired efferocytosis, accompanied by reactivation of PI3K and Rac1 in macrophages. Moreover, pharmacological agents targeting the PI3K/Rac1 pathway restored NETs-induced impairment in efferocytosis, leading to rapid wound healing. Raw264.7 cells exhibited elevated activation levels of PI3K and Rac1 when co-cultured with ACs in vitro. Nevertheless, this signaling activation was inhibited when cultured in a NETs-conditioned medium, leading to attenuated efferocytosis.

**Conclusions/interpretation:**

Targeting NETs and the PI3K/Rac1 pathway emerges as a potential therapeutic strategy to enhance healing in diabetic wounds by promoting macrophage efferocytosis.

**Supplementary Information:**

The online version contains supplementary material available at 10.1186/s12964-025-02092-4.

## Research in context

Previous studies have uncovered the presence of delayed wound healing, excessive NET formation, and diminished macrophage efferocytosis in diabetic wounds. Can these three phenomena be interrelated? This investigation revealed that macrophage efferocytosis in both in vitro and in vivo wound contexts is attenuated by NETs, an effect mediated through the PI3K/Rac1 pathway. Ultimately, this results in impaired wound healing in mice, suggesting a potential avenue for future clinical treatment of refractory diabetic wounds, that is, intervening in either the NETs or PI3K/Rac1 signaling pathways.

**Supplementary Information:**

The online version contains supplementary material available at 10.1186/s12964-025-02092-4.

## Introduction

Diabetes mellitus, a chronic condition characterized by elevated blood glucose levels, has seen a significant rise in incidence alongside improved living standards and lifestyle changes [1–3]. Among its numerous complications, impaired skin wound healing is notably prevalent, particularly in diabetic foot ulcers, which often lead to limb amputations [1, 4]. In addition to the systemic factors, the local wound environment in diabetes is marked by the activation of stress-responsive transcription factors and the production of apoptotic cells (ACs) [4, 5]. The efficient clearance of these ACs is crucial for wound resolution and tissue remodeling. Impeded clearance can lead to a protracted inflammatory environment that further impairs healing [5, 6]. The interplay between these factors contributes to chronic, non-healing wounds in diabetic patients, emphasizing the need for targeted therapeutic strategies that address metabolic and immunological aspects of wound healing in diabetes.

Neutrophils serve a critical function as the primary leukocytes during the early phases of healing [7, 8]. In their role as antimicrobial agents, neutrophils produce extracellular traps through the expulsion of decondensed chromatin decorated with cytotoxic proteins, which are capable of ensnaring and eliminating microorganisms [7]. However, it is noteworthy that neutrophil extracellular traps (NETs) can also induce tissue damage. Research has indicated that alterations in the microenvironment, driven by elevated blood glucose levels in diabetic patients, facilitate NETosis at multiple levels [9, 10]. Although NETs are essential for pathogen clearance, their excessive presence can lead to persistent inflammation and tissue damage [11, 12]. Excessive NETs can trigger a cascade of cellular death events, increasing the ACs accumulation in the wound, which, in turn, exacerbates the inflammatory response [12–15]. This dual role of NETs highlights the delicate balance required in wound management, where both pathogen defense and tissue integrity must be carefully modulated to facilitate optimal healing outcomes.

During wound healing, the substantial influx of cells is counterbalanced by apoptosis and the subsequent efferocytosis of ACs [16]. Efferocytosis, the process of phagocytosing ACs, constitutes a fundamental aspect of the immune response [3]. This process is essential for resolving inflammation, playing a critical role in shielding tissues from the toxic contents of dying cells and preventing further tissue damage by promoting the production of anti-inflammatory cytokines and chemokines [16, 17]. Phagocytosis mediated by Fcγ receptors (FcR) in macrophages requires the activation of phosphatidylinositol 3-kinase (PI3K) and the Rho family GTPase Ras-related C3 botulinum toxin substrate 1 (Rac1). The activation of PI3K is crucial for the generation of 3′phosphoinositides (3′PI) on the phagosomal membrane, which orchestrates the later stages of phagocytosis essential for the internalization of large particles [18, 19]. Rac1 is involved in various pathways, including cytoskeletal reorganization, gene transcription, cell proliferation, and survival. Rac1 acts as a regulatory factor for actin assembly and is essential for membrane ruffling [18]. Research by Peter Beemiller et al. demonstrated that Rac1 remains active throughout phagosome formation and only deactivates upon phagosome closure [18]. An extensive study by Huan Tao and colleagues explained the intricate role of the PI3K/Rac1 pathway in the modulation of macrophage phagocytosis [20]. These findings highlighted the significance of the PI3K/Rac1 pathway in the orchestration of macrophage phagocytosis and also emphasized its potential as a therapeutic target for modulating immune responses.

We first hypothesized that excessive NET formation during the healing of diabetic wounds impairs macrophage efferocytosis, leading to the accumulation of uncleared ACs within the wounds, given the association between impaired wound healing in diabetes, NET formation, and defective macrophage efferocytosis. Furthermore, we proposed an additional hypothesis that excessive NET formation in diabetic wounds compromises the efferocytotic capacity of macrophages through the PI3K/Rac1 pathway, considering the established link between PI3K and Rac1 activation and efferocytosis. This study firstly provided the evidence that there is a link between excessive NET formation and impaired efferocytosis in diabetic wounds.

## Methods

### Cell culture and reagents

The mouse macrophage cell line, Raw264.7, was acquired from the Institute of Basic Medical Science of CAMS and PUMC. The cell cultures were maintained in the Dulbecco’s modified eagle medium (DMEM), supplemented with 10% fetal bovine serum (FBS), streptomycin (100 mg/mL), and penicillin (100 units/mL), under controlled conditions of 37 °C and a 5% CO2 atmosphere.

### Animal source and husbandry

Eight-week-old female SPF-grade C57BL/6 N mice with an average body weight of 18 ± 2 g (Sibeifu Company, Beijing) were subjected to an acclimation period of one week at 25 ºC under SPF-grade conditions, during which they were provided with ample water and standard rodent diet. The mice were used for experimentation after the acclimation. Ethical approval for the animal experiments conducted in this study was granted by the ethical committee of the Chinese PLA General Hospital.

### Isolation of murine bone marrow-derived neutrophils

The bilateral tibias and femurs were harvested from eight-week-old C57BL/6 N mice post-euthanasia. Bone marrow cavities were flushed with D-Hank’s buffer, which contained 0.38% sodium citrate. The resultant cell suspension was gently disaggregated and passed through a 40 μm cell strainer (Corning, USA) to acquire a single-cell suspension. Post-lysis of red blood cells, the single-cell suspension was washed in HBSS solution, further purified using a MACS-based neutrophil isolation kit (Milteyni Biotec, Germany) as per the manufacturer’s recommendations, and cultured in RPMI-1640 medium.

### Induction and isolation of NETs

Neutrophils derived from mouse bone marrow were seeded at a density of 1 × 10^6^ cells/mL onto poly-L-lysine-coated 12-well plates using RPMI-1640 basal medium. After adding 50 nM PMA, the cells were incubated for 4 h at 37 °C with 5% CO_2_, thereby initiating the formation of NETs. After removal of the supernatant, the NET monolayer was detached from the culture surface using ice-cold PBS and then centrifuged at 400 × g for 5 min at 4 °C to pellet cell debris. The cell-free supernatant was pooled and subsequently centrifuged at 15,000 × g for 15 min at 4 °C to pellet DNA. The DNA pellet was resuspended in PBS to a volume equivalent to 100 µL per 1 × 10^7^ neutrophils, thus obtaining cell-free NETs. For in vitro intervention experiments, 20 µL of NET-containing PBS was mixed with each 1 mL of medium to prepare the conditioned medium.

### Quantification of NETs

NETs were isolated according to previously established protocols. The extracted extracellular DNA was supplemented with the fluorescent dye Sytox green (1:1000, Biolab, China) and subsequently incubated in the dark for 15 min. The quantity of extracellular DNA was assessed by measuring the average fluorescence intensity using an enzyme-linked immunosorbent assay reader.

### Establishment of the streptozotocin (STZ)-induced diabetic mouse model

A buffer solution was prepared by mixing 100 mM of citric acid and 100 mM of sodium citrate in a 1:1 ratio. Additionally, 1 g of STZ per 100 mL was added to the buffer solution. The mice were intraperitoneally injected with the previously prepared STZ solution at a dosage of 150 mg STZ per kg of body weight after a 24-h fasting period during which access to water was unlimited. Following the injection, the mice were given sufficient access to water and food. Successful model induction was confirmed by maintaining fasting blood glucose levels at or above 10 mmol/L for a continuous week after a six-day follow-up observation period.

The dosage and timing for intraperitoneal injection of the reagent were as follows: Cl-Ad (NETs inhibitor; MCE, China) at 25 mg/kg dose administered on day 1 before wound modeling and days 2 and 5 post-wound. The dosages for subcutaneous injection of the reagents were as follows: NSC23766 (Rac1 inhibitor; MCE, China) at 4 mg/kg; ML-099 (Rac1 agonist; MCE, China) at 20 mg/kg; wortmannin (PI3K inhibitor; MCE, China) at 1.2 mg/kg; and 740Y-P (PI3K agonist; MCE, China) at 10 mg/kg. All subcutaneous injections were administered on days 0, 3, and 5 post-wound.

### Wound healing model

All experimental procedures complied with relevant international regulations as outlined in the guide for the care and use of laboratory animals. The study was approved by the Institutional Animal Care and Use Committee for animal experiments. After anesthesia induction in each STZ-induced diabetic mouse, the dorsal region of the mouse was meticulously prepared for wound creation. Initially, the fur was gently shaved using an electric clipper to ensure a clean and hair-free area. This was followed by a thorough disinfection with 70% ethanol, which was applied to the skin surface to sterilize the area and minimize the risk of infection. After the skin had been properly prepared, a sterile punch biopsy needle with an 8 mm diameter was carefully inserted into the dermis to create a standardized full-thickness excisional wound. Post-wound creation, the mice were monitored closely, and the progression of wound healing was meticulously documented through digital images taken at defined intervals.

The sample size was determined using the formula for degrees of freedom (DF): DF = N - K = K*n - K = K (n-1), where DF represents the degrees of freedom, N is the total number of experimental animals, K is the number of experimental groups, and the n is the number of animals per group. DF often ranges from 10 to 20.

### In vivo macrophages depletion

To achieve macrophage depletion, C57BL/6 N mice were intraperitoneally injected with 200 µL of a macrophage-depleting solution (Clodronate Liposomes, Yeasen, China) beginning one day before the adoptive transfer of macrophages. The efficacy of macrophage depletion was assessed via flow cytometry. This analysis quantitatively verified the reduction in macrophage populations, confirming the successful execution of the macrophage-depleting strategy.

### Isolating macrophages from wounds

The excised mouse skin tissues were finely minced in PBS and then incubated for 30 min at 37 ℃ in a tissue digestion solution composed of 1 mg/mL type IV collagenase and 0.2 mg/mL DNase I in PBS. The digested tissue was gently dissociated and filtered through a 40 μm cell strainer (Corning, USA) to obtain a single-cell suspension. After two PBS washes, the single-cell suspension was subjected to macrophage purification using the MagniSort™ mouse F4/80 positive selection kit (Thermo Fisher, USA). The efficacy of isolation was assessed via flow cytometry.

### In vivo adoptive transfer of macrophages

To facilitate the adoptive transfer of macrophages, macrophages were isolated from the skin wounds of C57BL/6 N mice by using a MagniSort™ mouse F4/80 positive selection kit (Thermo Fisher, USA) following the manufacturer’s protocol. The recipient mice were given a single subcutaneous injection of macrophages (1 × 10^5^) resuspended in 100 µL of ice-cold HBSS on the second day of wound modeling. The purpose of this adoptive transfer was to introduce macrophages into the recipient mice to facilitate subsequent investigation and analysis.

### Assay of in vivo efferocytosis

Following the induction of a full-thickness excisional wound on the dorsal region of eight-week-old female diabetic mice, the animals were humanely euthanized after a predetermined treatment interval. Subsequently, the wound beds were carefully excised and preserved in polyformaldehyde, followed by paraffin embedding and sectioning. The sections were then precisely stained with TUNEL reagent to identify ACs, and an immunofluorescent stain was applied using F4/80 antibody (5 µg/mL, Abcam, USA) to specifically target macrophages. TUNEL + nuclei were quantified according to their association or lack of association with F4/80 macrophages to evaluate in situ efferocytosis. The degree of macrophage efferocytosis is represented by the ratio of “free” to “macrophage-associated” ACs.

### Induction of ACs

Neutrophils derived from murine bone marrow were exposed to 254 nm ultraviolet irradiation for 15 min, followed by incubation under standard cell culture conditions for 2 to 3 h. This procedure typically yields more than 85% of Annexin V-positive cells. The ACs were rinsed once with serum-free DMEM, resuspended at a concentration of 2 × 10^7^ cells/mL in Diluent C (Sigma-Aldrich, USA), and incubated for 3 min with 4 mL of Diluent C containing concentrated PKH67 green dyes (Sigma-Aldrich, USA). Subsequently, the cells were prepared for experimental use after being rinsed twice with DMEM supplemented with 10% heat-inactivated FBS.

### In vitro efferocytosis assay

Macrophages derived from either wound tissue or the Raw264.7 cell line were seeded into 24-well plates at a density of 0.2 × 10^6^ cells per well. Once the cells had adhered to the plate, they were washed twice with PBS and subsequently incubated with 500 µL of DiI red staining solution at 37 °C. Following a 20-min incubation period, the macrophages were washed thrice with PBS. PKH67-labeled ACs were then added to the macrophages at a 5:1 (ACs to macrophages) ratio. After 45 min, the cells were again washed three times with PBS to eliminate unbound ACs. Subsequently, the macrophages were fixed in 4% formaldehyde for 20 min, washed thrice with PBS, and then imaged using a Leica inverted fluorescence microscope (DMI6000B). During Western blotting, the staining and fixation steps were omitted for both macrophages and ACs.

The reagent treatment concentration was as follows: Cl-Amidine (NETs inhibitor) at 20 µM; NSC23766 (Rac1 inhibitor) at 100 µM; ML-099 (Rac1 agonist) at 25 nM; wortmannin (PI3K inhibitor) at 10 nM; and 740Y-P (PI3K agonist) at 30 µM. The reagents were added to the conditioned medium simultaneously at the start of the in vitro cell burial assay.

### Plasmid construction and transfection

For gene intervention experiments, macrophages were transfected with plasmids overexpressing Rac1Q61L, PI3K or their corresponding control vectors using Lipofectamine 3000 (Invitrogen) according to the manufacturer’s instructions. Briefly, cells were seeded in 6-well plates and grown to 70–80% confluence. Plasmid DNA and Lipofectamine 3000 reagent were mixed in Opti-MEM medium and incubated for 15 min before adding to the cells. After 48 h of transfection, cells were harvested and used for efferocytosis assays.

### Flow cytometry

For flow cytometry analysis, cellular suspensions were washed once with PBS and permeabilized using eBioscience™ Foxp3/transcription factor staining buffer set (catalog NO.00-5523-00), following the manufacturer’s instructions. Conjugated antibodies included anti-CD11b (1:1000, Abcam) and anti-F4/80 (1:50, Abcam) antibody were added and incubated for 1 h at room temperature. Appropriate isotype controls were employed to interpret the results accurately. Cells were first gated based on FSC/SSC to exclude debris, followed by selection of CD11b + cells, and macrophages were identified by F4/80 expression within the CD11b + population.

### Western blot analysis

Protein concentration in cell lysates was determined using the bicinchoninic acid protein assay. Subsequently, proteins were separated by 10% sodium dodecyl sulfate-polyacrylamide gel electrophoresis and transferred onto polyvinylidene difluoride membranes (Millipore, USA). The membranes were blocked with 5% BSA for 60 min at room temperature, followed by incubation with primary antibodies overnight at 4 ℃. Subsequently, the membranes were washed three times for 10 min each with Tris-buffered saline containing 0.1% Tween 20 and then incubated for 1 h at room temperature in a blocking solution containing horseradish peroxidase-conjugated secondary antibodies.

Each experiment was repeated three times. The proteins were visualized using the LAS-3000 luminescent image analyzer (FujiFilm, Tokyo, Japan).

### Statistical analyses

All statistical analyses were performed using GraphPad Prism 8 software (San Diego, CA, USA). Data were represented as mean ± SEM from at least three independent experiments. An unpaired two-tailed Student’s t-test was used to compare two groups, while one-way ANOVA was applied for comparisons involving three or more groups. A *p*-value less than 0.05 was considered statistically significant, with symbols indicating levels of significance (*P < 0.05, **P < 0.01, ***P < 0.001).

## Results

### Neutrophils extracellular traps impair efferocytosis in vitro and in vivo

**Fig. 1:**
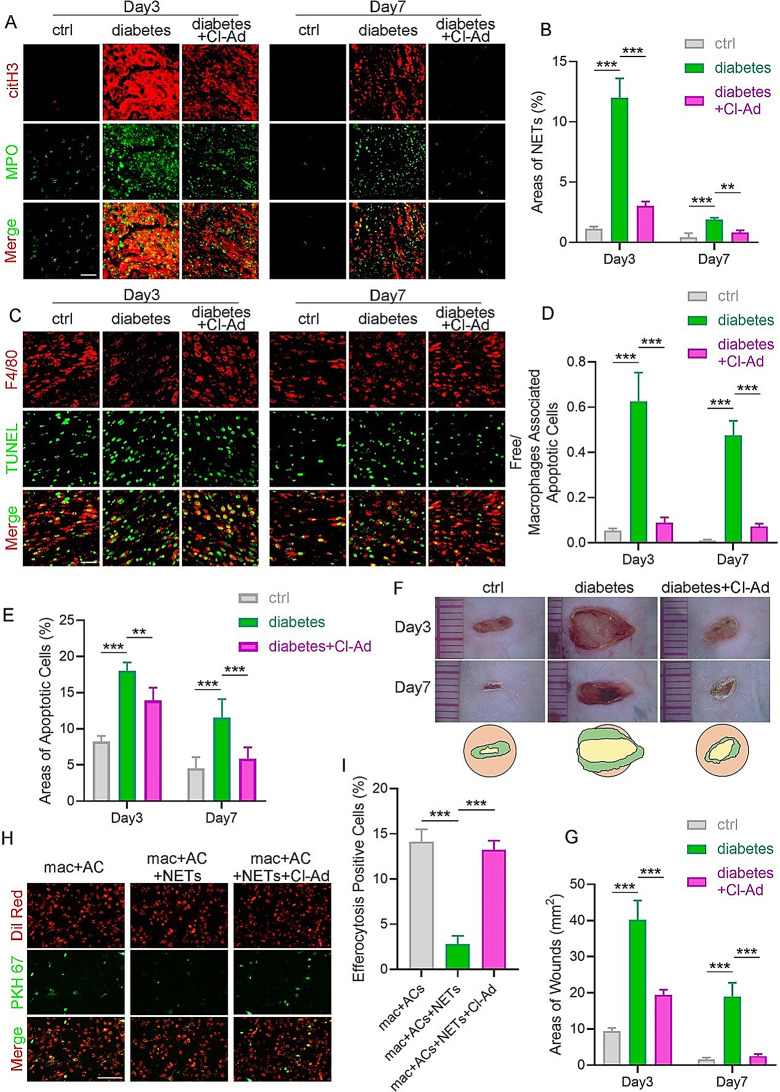
NETs examination and efferocytosis assays in vitro and *in vivo.* (**A**) Representative immunofluorescence images of citH3 and MPO, the main components of NETs, in wounds of normal, diabetic, and Cl-Ad-treated diabetic mice. *N* = 5 per group. Scale bar, 25 μm. (**B**) The area of colocalization of citH3 and MPO was defined as the area of NETs. This is a graph of the NETs quantitative evaluation in (**A**). (**C**) Representative immunofluorescence images of macrophages (F4/80+) and ACs (TUNEL+) in the wounds of normal, diabetic, and Cl-Ad-treated diabetic mice. *N* = 5 per group. Scale bar, 25 μm. (**D**) The value of “free/macrophages-associated ACs” was defined as the strength of efferocytosis. A smaller value indicates greater efferocytosis intensity. This is a graph of the efferocytosis quantitative evaluation in (**C**). (**E**) Quantitative evaluation of ACs areas in (**C**). (**F**) The gross view of wounds in the control, diabetic, and Cl-Ad-treated diabetic groups was observed on days 3 and 7. (**G**) Measuring the extent of wound healing in (**F**). (**H**) Fluorescent images demonstrating PKH67-labeled ACs were efferocytosed by Raw264.7 cells stained with Dil. Scale bar, 50 μm. (**I**) Quantitative evaluation of the findings in (**H**). Data were represented as mean ± SEM; ns indicated no significant difference; *P < 0.05; **P < 0.01; ***P < 0.001

Immunofluorescence staining was initially performed to investigate the relationship between NETs and the efferocytosis of ACs by macrophages in wounds of diabetic mice. Compared to normal controls, wounds of diabetic mice exhibited increased levels of NETs, consistent with previous findings (Fig. 1A-B). However, diabetic wounds revealed diminished phagocytic activity, which was evident by fewer TUNEL + ACs internalized by F4/80 + macrophages (Fig. 1C-D). To further elucidate whether the lower phagocytic activity in macrophages was associated with the abundant NET level in diabetic wounds, the NET level was downregulated on the wound site, and then efferocytosis activity was detected. Peptidylarginine deiminase 4 (PAD4) is a histone-modifying enzyme highly expressed in neutrophils, which plays a critical role in NET formation. Consequently, we used Cl-Ad, an oral active PAD inhibitor, to stop the NET formation and to investigate the link between NET formation and efferocytosis. No differences in macrophage infiltration of wound were observed in diabetic mice treated with Cl-amidine (Fig. S1A-B), suggesting that recruitment of these cells was not blocked by Cl-amidine. As demonstrated in Fig. 1A-B, NET inhibition induced by Cl-Ad resulted in decreased NET formation in diabetic wounds. Notably, the decrease in NET level restored the efferocytosis activity of macrophages in diabetic wounds (Fig. 1C-D). Consequently, the expanded area of ACs in the wounds of diabetic mice was reduced following NETs inhibition (Fig. 1C-E), accompanied by accelerated wound healing (Fig. 1F-G). To further validate these results in vitro, Raw264.7 macrophages were treated with NETs, and their efferocytosis activity was detected. The results demonstrated that Raw264.7 macrophages phagocytized less PKH67-labeled ACs after the intervention of NETs, revealing that efferocytosis was impaired in the presence of NETs (Fig. 1H-I). Nevertheless, this impairment was rescued by Cl-Ad-induced NET inhibition (Fig. 1H-I). Taken together, these findings indicated that NETs in diabetic mouse wounds contribute to defective efferocytosis of macrophage.

### NETs in diabetic mice have a persistent effect on the impaired efferocytosis of macrophages

**Fig. 2:**
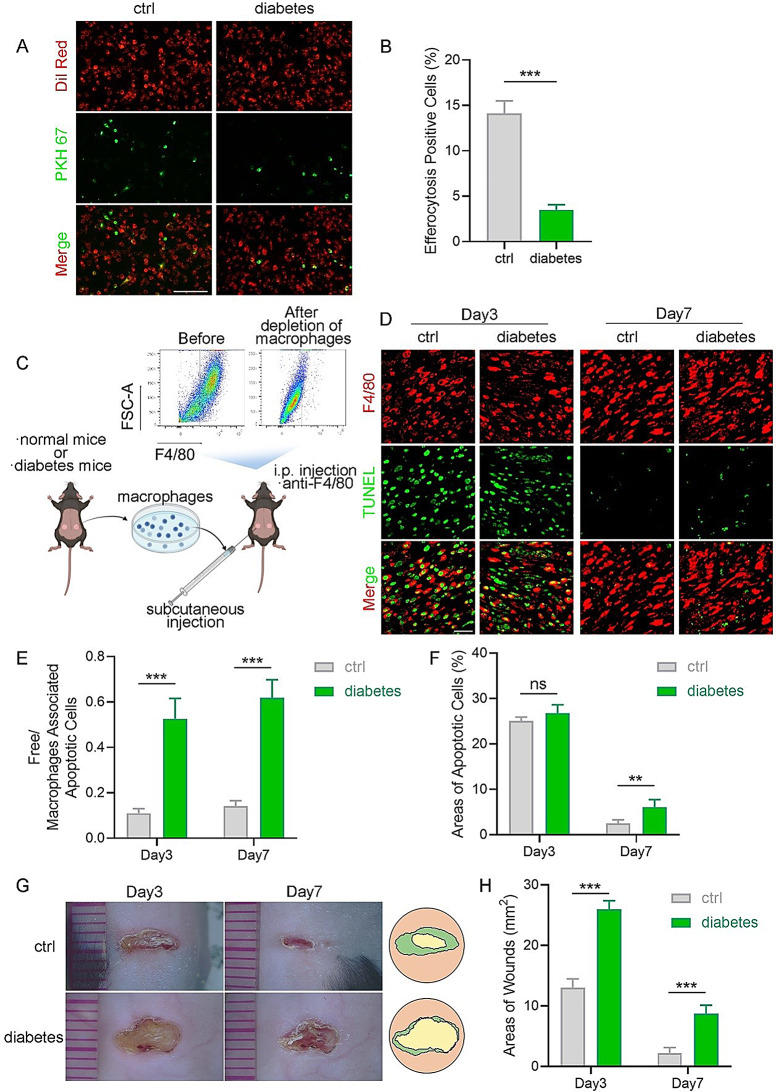
Efferocytosis assays in vitro and macrophages adoptive transplantation assays. (**A**) Fluorescent images demonstrating PKH67-labeled ACs being efferocytosed by Dil-labeled macrophages derived from normal and diabetic mice. Scale bar, 50 μm. (**B**) Quantitative evaluation of the findings in (**A**). (**C**) Schematic diagram illustrating host macrophages ADT to macrophages-depleted normal mice. (**D**) Representative immunofluorescence images of macrophage efferocytosis in wounds of normal mice that received macrophages ADT from normal wounds and diabetic wounds. *N* = 5 per group. Scale bar, 25 μm. (**E**-**F**) Quantitative evaluation of the findings in (**D**). (**G**) The gross view of wounds in the control and diabetic macrophage-transferred groups was observed on days 3 and 7. (**H**) Measuring the extent of wound healing in (**G**). Data were represented as mean ± SEM; ns indicated no significant difference; *P < 0.05; **P < 0.01; ***P < 0.001

Macrophages were isolated from the wound sites of both STZ-induced diabetic and normal mice to assess the persistent impairment of macrophage efferocytosis induced by NETs. After incubation with ACs, macrophages isolated from diabetic wounds exhibited compromised efferocytosis, compared to those isolated from healthy wounds, displaying typical efferocytosis (Fig. 2A-B). To further complicate this matter, mouse macrophages were depleted using a macrophage-depleting solution (Clodronate Liposomes, Yeasen, China), and then adoptively transferred (ADT) macrophages that were isolated from normal or diabetic wounds (Fig. 2C). Surprisingly, impaired efferocytosis and increased ACs areas were observed in macrophage-depleted mice receiving subcutaneous injections of macrophages from diabetic mice (Fig. 2D-F). Concurrently, the group of mice that received diabetic macrophages exhibited delayed wound healing (Fig. 2G-H). These results further confirmed the sustained impairment of efferocytosis caused by NETs in diabetic mice.

### NETs impair efferocytosis in vitro by suppressing Rac1 activation

**Fig. 3:**
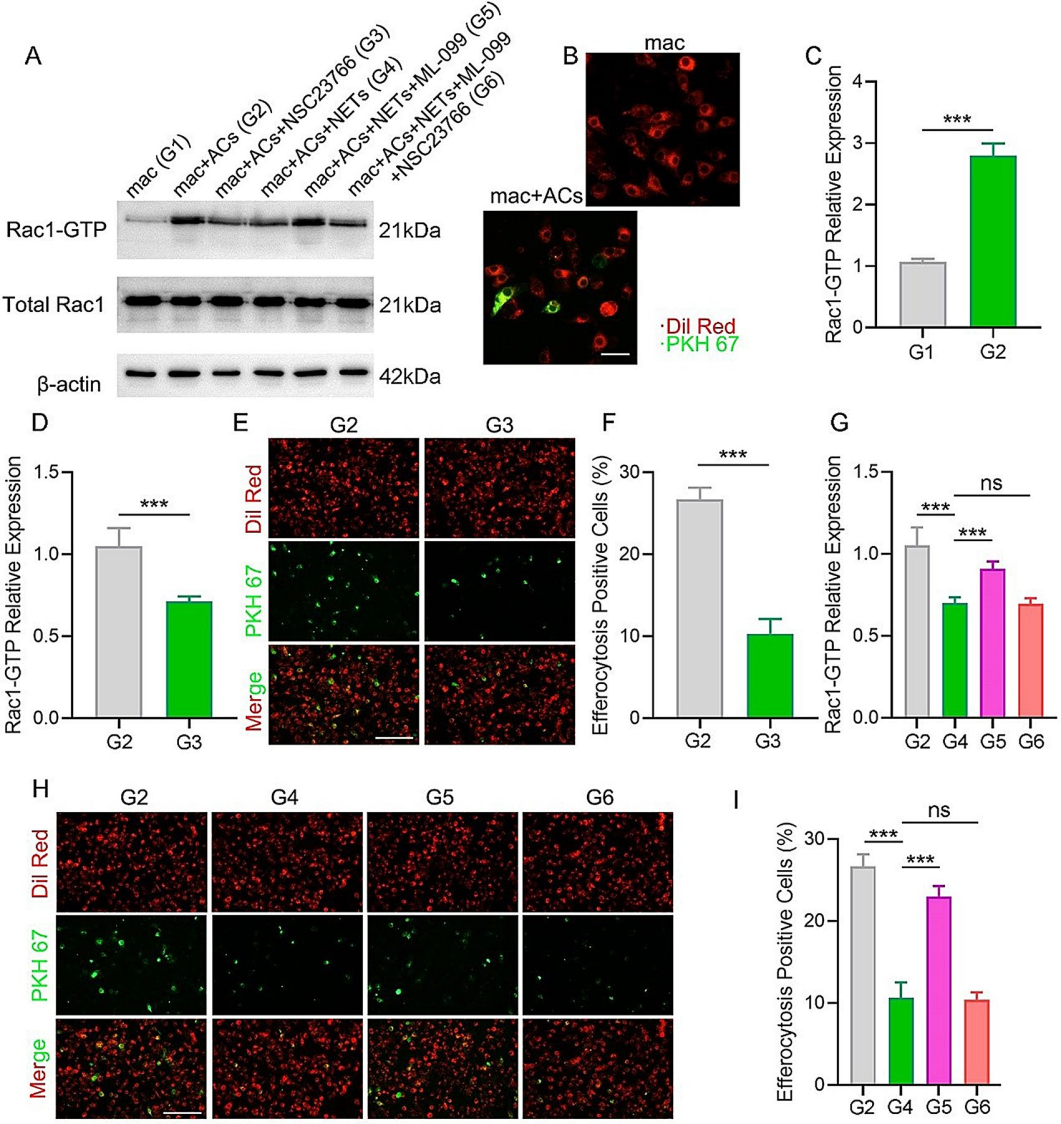
Exploring the role of NETs and Rac1-GTP in efferocytosis *in vitro.* (**A**) Rac1-GTP and total Rac1 expression levels in Raw264.7 cells were analyzed using Western blotting. (**B**) Raw264.7 cells were either treated with ACs or left untreated, and then the macrophages were washed to eliminate the unbound ACs for efferocytosis examination. Scale bar, 10 μm. (**C**) Protein expression levels of Rac1-GTP in Raw264.7 cells were quantified using ImageJ software following treatment with ACs. (**D**) Protein expression levels of Rac1-GTP during efferocytosis were quantified following treatment with Rac1 activation inhibitor NSC23766. (**E**) Fluorescent images demonstrating Raw264.7 cells undergoing efferocytosis under NSC23766-treated or untreated conditions. Scale bar, 50 μm. (**F**) Quantitative evaluation of the findings in (**E**). (**G**) Protein expression levels of Rac1-GTP during efferocytosis were quantified following treatment with NETs, NETs + ML-099, NETs + ML-099 + NSC23766, and the untreated group. (**H**) Fluorescent images illustrating Raw264.7 cell efferocytosis from the control group and NETs, NETs + ML-099, NETs + ML-099 + NSC23766 treated groups. Scale bar, 50 μm. (**I**) Quantitative evaluation of the findings in (**H**). G1, G2, G3, G4, G5, and G6 indicate the macrophagy (mac), mac + ACs, mac + ACs + NSC23766, mac + ACs + NETs, mac + ACs + NETs + ML-099, and mac + ACs + NETs + ML-099 + NSC23766, respectively. Data were represented as mean ± SEM; ns indicated no significant difference; *P < 0.05; **P < 0.01; ***P < 0.001

Efferocytosis is tightly regulated in both prokaryotic and eukaryotic cells through evolutionarily conserved signaling pathways that converge on Rac1 activation, leading to cytoskeletal rearrangements necessary for engulfment [21]. As a result, this study investigated Rac1 activation in Raw264.7 cells and assessed the effects of pharmacological inhibition of Rac1 on efferocytosis. Upon incubation with ACs in vitro, Rac1 activation, detected as Rac1-GTP, was significantly elevated in macrophages (Fig. 3A-C). Then, NSC23766, a specific Rac1 inhibitor, was used to induce Rac1 inhibition to investigate the role of Rac1 in macrophage efferocytosis. The results indicated that NSC23766 significantly reduced the levels of active Rac1 in macrophages (Fig. 3A and D) and impaired macrophage efferocytosis of ACs (Fig. 3E-F). These results indicated that macrophage efferocytosis is regulated by Rac1 activation.

To investigate whether NETs regulate efferocytosis by modulating Rac1 activation, we first measured Rac1 activation during efferocytosis in the presence of NETs. As demonstrated in Fig. 3A and G, Rac1 activation was reduced during efferocytosis when treated with NETs as compared to the untreated group. Activation of Rac1 with ML-099 (Rac1 agonist) elevated Rac1-GTP levels in macrophages exposed to NETs (Fig. 3A and G). Notably, ML-099 treatment restored defective efferocytosis caused by NETs (Fig. 3H-I). The finding that pharmacological activation of Rac1 rescues efferocytosis in the presence of NETs supports the hypothesis that Rac1 is a key mediator of NETs-regulated efferocytosis. Since ML-099 likely activates multiple signaling pathways, the effects of the specific Rac1 inhibitor, NSC23766, on macrophage efferocytosis were also assessed. NSC23766 significantly decreased Rac1 activation in macrophages exposed to NETs and ML-099 and reduced efferocytosis to levels comparable to those in macrophages incubated with NETs alone (Fig. 3G-I). Moreover, we made macrophages that overexpress dominant-positive mutant Rac1Q61L, which can overexpress Rac1 in a constitutively GTPase-activated state. As shown in Fig. S2A-B, increased efferocytosis occurred in macrophages overexpressing Rac1Q61L. These results demonstrated that NETs downregulate macrophage efferocytosis by deactivating Rac1.

### NETs impair efferocytosis by suppressing the activation of Rac1 of macrophages in diabetic wounds

**Fig. 4:**
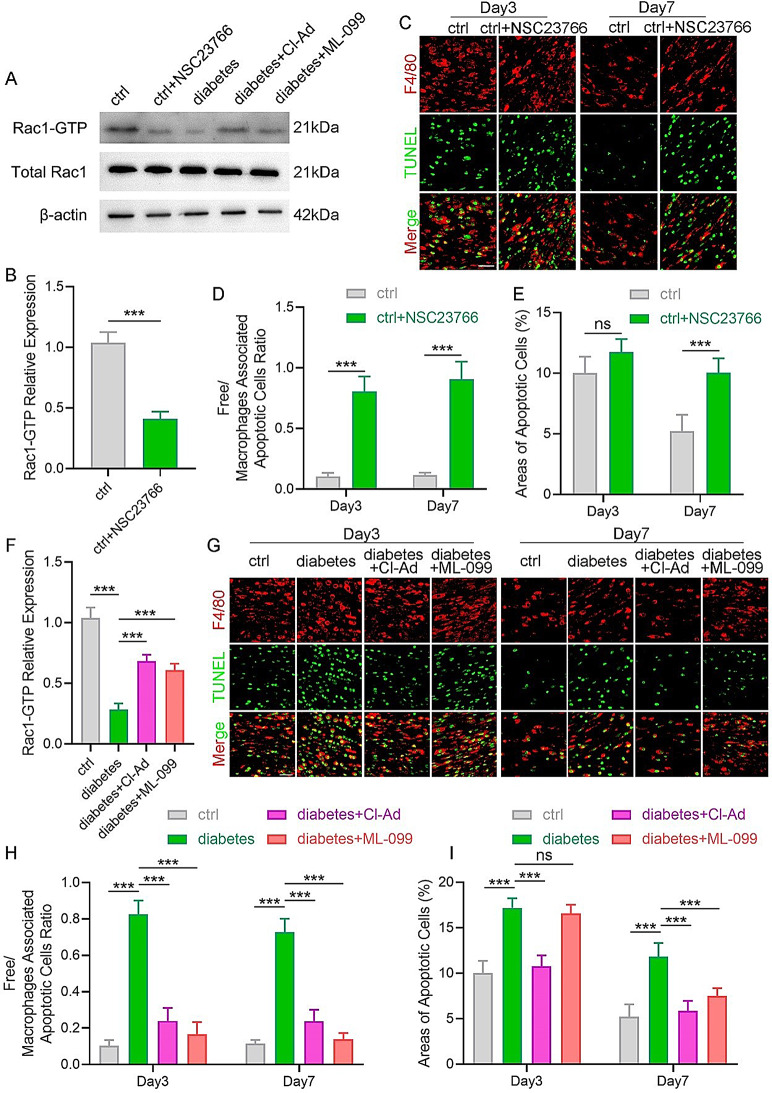
Exploring the role of NETs and Rac1-GTP in efferocytosis *in vivo.* (**A**) Rac1-GTP and total Rac1 expression levels of macrophages in mouse wounds on day 3 were analyzed using Western blotting. *N* = 5 per group. (**B**) Macrophage protein expression levels of Rac1-GTP in the wounds of control and NSC23766-treated normal mice groups were quantified. (**C**) Representative immunofluorescence images of macrophage efferocytosis in the wounds of control and NSC23766-treated normal mice groups. *N* = 5 per group. Scale bar, 25 μm. (**D-E**) Quantitative evaluation of the findings in (**C**). (**F**) Macrophage protein expression levels of Rac1-GTP in wounds were quantified from normal, diabetic, and diabetic mice treated with Cl-Ad and ML-099 groups. (**G**) Representative immunofluorescence images of macrophage efferocytosis in wounds of normal, diabetic, and diabetic mice treated with Cl-Ad and ML-099 groups. *N* = 5 per group. Scale bar, 25 μm. (**H-I**) Quantitative evaluation of the findings in (**G**). Data were represented as mean ± SEM; ns indicated no significant difference; *P < 0.05; **P < 0.01; ***P < 0.001

The role of Rac1 in regulating efferocytosis during normal wound healing was also explored. NSC23766 treatment effectively downregulated the levels of Rac1-GTP in wound-derived macrophages compared to the untreated group (Fig. 4A-B). Concurrently, a decrease in efferocytosis and an increase in the area of ACs was observed in the NSC23766-treated group (Fig. 4C–E). A delay in wound healing was also noticed in the NSC23766-treated group (Fig. S3A-B). Accordingly, the finding that macrophage efferocytosis is regulated by Rac1 activation in mice wounds aligns with the in vitro results.

To further confirm Rac1 as a mediator of NETs-regulated efferocytosis in diabetic wounds, NETs inhibition was induced by using Cl-Ad. Then, the levels of Rac1 activation were assessed in wound-derived macrophages. Reduced Rac1-GTP levels were observed in diabetic wound macrophages, which were restored by NET inhibition (Fig. 4A and F). Moreover, subcutaneous administration of ML-099 (Rac1 agonist) in diabetic mouse wounds, which did not affect NET formation (Fig. S3C-D), suggested that Rac1 activation functions downstream of NETs in vivo. Consistent with the in vitro model, ML-099 intervention in diabetic wounds resulted in increased Rac1-GTP levels in macrophages (Fig. 4A and F), enhancement of macrophage efferocytosis (Fig. 4G and H), decreased ACs areas (Fig. 4G and I), and rapid wound healing (Fig. S3E-F), confirming that NETs impaired efferocytosis by suppressing Rac1 activation of macrophages, thus delayed wound healing was noted in diabetic mouse wounds.

### NETs affect efferocytosis via PI3K/Rac1 signaling in vitro

**Fig. 5:**
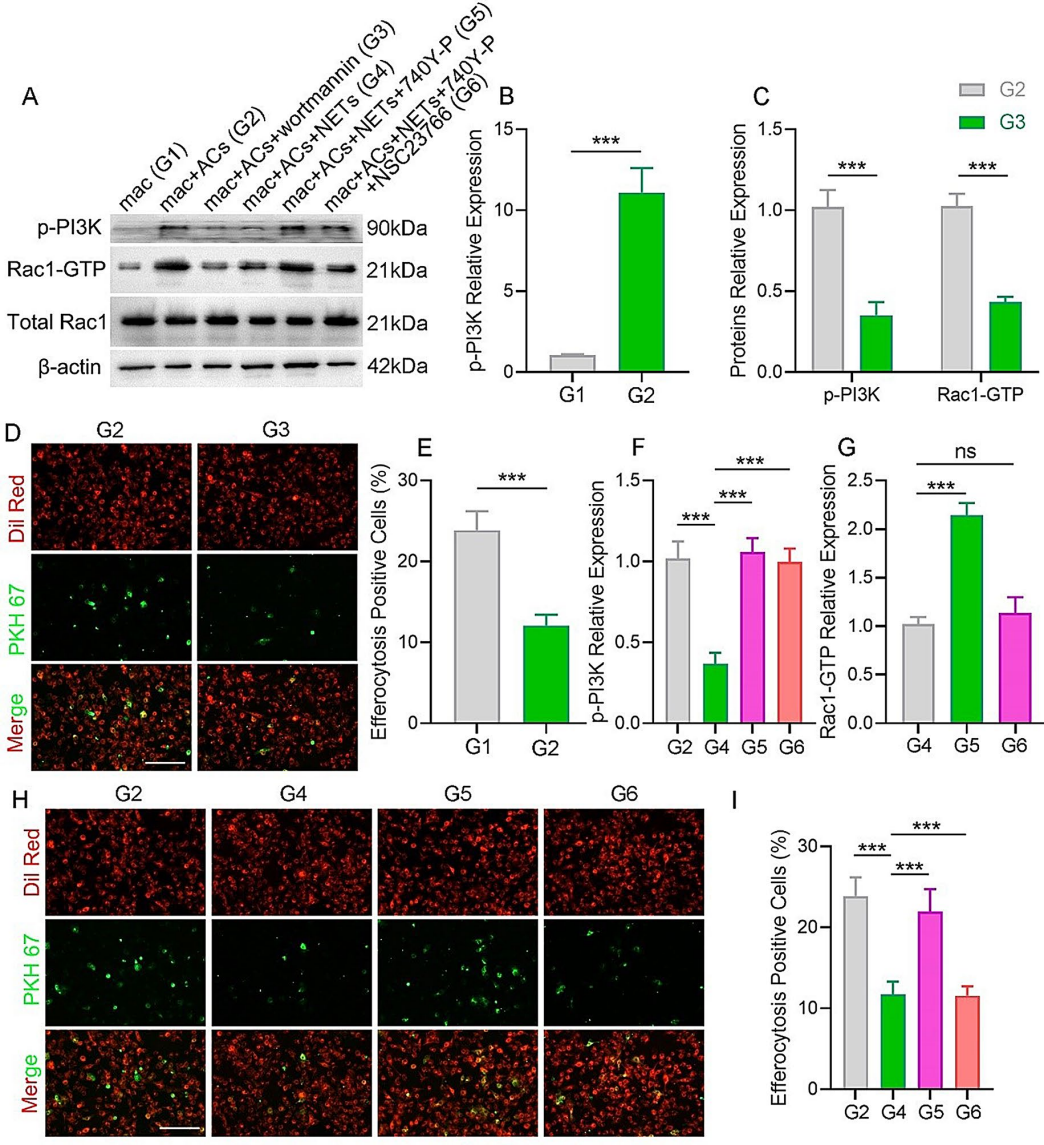
Exploring the role of NETs, p-PI3K, and Rac1-GTP in efferocytosis *in vitro.* (**A**) The expression levels of p-PI3K, Rac1-GTP, and total Rac1 in Raw264.7 cells were analyzed using Western blotting. (**B**) Protein expression levels of p-PI3K in Raw264.7 cells were quantified for the control and ACs-treated groups. (**C**) Protein expression levels of p-PI3K and Rac1-GTP in Raw264.7 cells during efferocytosis were quantified for the control and wortmannin-treated groups. (**D**) Fluorescent images demonstrating Raw264.7 cells undergoing efferocytosis under wortmannin-treated or untreated conditions. Scale bar, 50 μm. (**E**) Quantitative evaluation of the findings in (**D**). (**F**) Protein expression levels of p-PI3K in Raw264.7 cells during efferocytosis were quantified for the control, NETs, NETs + 740Y-P, and NETs + 740Y-P + NSC23766 treated groups. (**G**) Protein expression levels of Rac1-GTP in Raw264.7 cells during efferocytosis were quantified for NETs, NETs + 740Y-P, and NETs + 740Y-P + NSC23766 treated groups. (**H**) Fluorescent images demonstrating Raw264.7 cell efferocytosis under NETs, NETs + 740Y-P, and NETs + 740Y-P + NSC23766 treated or untreated groups. Scale bar, 50 μm. (**I**) Quantitative evaluation of the findings in (**H**). G1, G2, G3, G4, G5, and G6 indicate the mac, mac + ACs, mac + ACs + wortmannin, mac + ACs + NETs, mac + ACs + NETs + 740Y-P, and mac + ACs + NETs + 740Y-P + NSC23766, respectively. Data were represented as mean ± SEM; ns indicated no significant difference; *P < 0.05; **P < 0.01; ***P < 0.001

Previous studies have indicated that PI3K activity is essential for phagosome formation [22]. Consequently, PI3K phosphorylation and its interaction with Rac1 activation were investigated in the Raw264.7 cell line of murine macrophages. PI3K phosphorylation was significantly enhanced in Raw264.7 macrophages incubated with ACs (Fig. 5A-B). This result indicated that PI3K may be involved in macrophage efferocytosis. In the process of macrophage phagocytosis, the generation of 3′PI on the phagosomal membrane by PI3K is instrumental in orchestrating the later stages of phagocytosis that are essential for the internalization of large particles [18–20]. During this process, Rac1 functions as a regulatory factor for actin assembly, which is crucial for membrane ruffling [18, 20]. Accordingly, it was hypothesized that the coordinated action of PI3K and Rac1 is fundamental to the efficient execution of phagocytosis. To verify this hypothesis, we used wortmannin, a PI3K inhibitor that covalently binds to the p110 subunit of PI3K, to induce PI3K inhibition. As expected, decreased Rac1-GTP levels were observed following wortmannin inhibition of PI3K in macrophages, which identified that Rac1 is a downstream target of PI3K (Fig. 5A and C). Concurrently, wortmannin-treated macrophages displayed reduced efferocytosis activity (Fig. 5D-E).

The potential of NETs to mediate efferocytosis via the PI3K/Rac1 pathway was assessed in vitro. PI3K and Rac1-GTP phosphorylation was diminished in the NETs-treated group during efferocytosis (Fig. 5A, F, and G), as compared to the NETs-free group. Interestingly, 740Y-P was used to activate PI3K (Fig. 5A and F), and the results revealed that 740Y-P rescued the NETs-induced deactivation of Rac1-GTP (Fig. 5A and G) and restored efferocytosis (Fig. 5H-I). Additionally, overexpression of PI3K in macrophages reversed efferocytosis inhibition following incubation with NETs (Fig. S4A-B). Furthermore, NSC23766 was used to inhibit the elevated effect of 740Y-P on Rac1-GTP. It was revealed that NSC23766 significantly reversed the rescue effect of 740Y-P on efferocytosis (Fig. 5H-I). When combined, these findings indicated that NETs regulate efferocytosis via the PI3K/Rac1 signaling pathway in vitro.

### NETs affect efferocytosis via PI3K/Rac1 signaling in diabetic mouse wounds

**Fig. 6:**
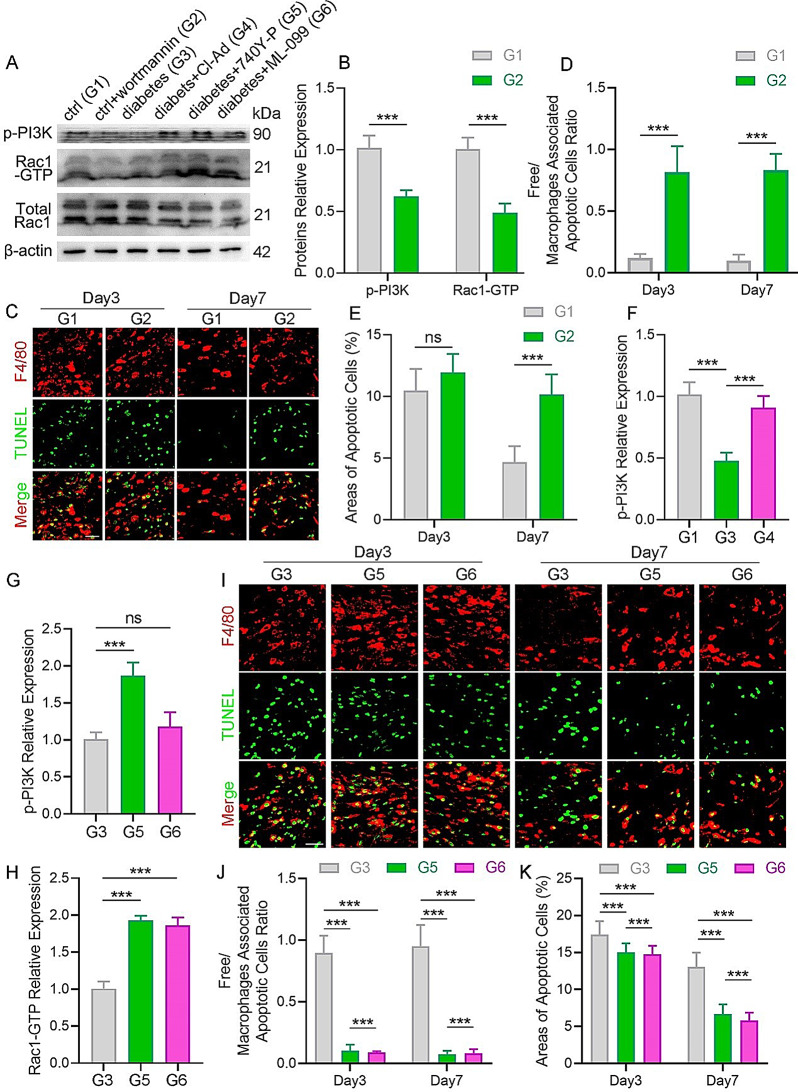
Exploring the role of NETs, p-PI3K, and Rac1-GTP in efferocytosis *in vivo.* (**A**) Macrophages p-PI3K, Rac1-GTP, and total Rac1 expression levels in mouse wounds on day 3 were analyzed using Western blotting. *N* = 5 per group. (**B**) Macrophage protein expression levels of p-PI3K and Rac1-GTP in the wounds of normal and wortmannin-treated normal mice were quantified. (**C**) Representative immunofluorescence images of macrophage efferocytosis in wounds of normal and wortmannin-treated normal mice. *N* = 5 per group. Scale bar, 25 μm. (**D-E**) Quantitative evaluation of the findings in (**C**). (**F**) Macrophage protein expression levels of p-PI3K in wounds of normal, diabetic, and Cl-Ad-treated diabetic mice were quantified. (**G-H**) Macrophage protein expression levels of p-PI3K and Rac1-GTP in diabetic wounds, 740Y-P, and ML-099 treated diabetic mice were quantified. (**I**) Representative immunofluorescence images of macrophage efferocytosis in wounds of normal, diabetic, and 740Y-P treated diabetic mice. *N* = 5 per group. Scale bar, 25 μm. (**J-K**) Quantitative evaluation of the findings in (**I**). G1, G2, G3, G4, G5, and G6 indicate the control, control + wortmannin, diabetic, diabetic + Cl-Ad, diabetic + 740Y-P, and diabetic + ML-099, respectively. Data were represented as mean ± SEM; ns indicated no significant difference; *P < 0.05; **P < 0.01; ***P < 0.001

Then, the effect of macrophage PI3K phosphorylation on efferocytosis in mouse wounds was investigated. Wortmannin was injected intraperitoneally to induce PI3K inhibition, and the inhibition effect on wound macrophages was detected. Following wortmannin treatment, the levels of p-PI3K were reduced in wound macrophages (Fig. 6A-B). Meanwhile, it was found that Rac1-GTP levels were simultaneously decreased (Fig. 6A-B). Concurrently, a decreased occurrence of efferocytosis (Fig. 6C-D), increased ACs area (Fig. 6C and E), and delayed wound healing (Fig. S5A-B) was observed in wortmannin-treated normal mouse wounds. As a result, the in vitro results are consistent with the findings that macrophage efferocytosis on the wound site is regulated via the PI3K/Rac1 signaling pathway, which contributes to wound healing.

To verify that NETs within diabetic wounds inhibit macrophage efferocytosis by downregulating the PI3K/Rac1 pathway, the levels of p-PI3K were initially evaluated in diabetic and normal mouse wound macrophages. As demonstrated in Fig. 6A and F, a decrease in p-PI3K levels was observed in diabetic wound macrophages compared to the normal wound macrophages. Notably, the decreased trend in p-PI3K levels was restored following the treatment with Cl-Ad, a NET inhibitor (Fig. 6A and F). To further complicate this matter, 740Y-P or ML-099 was used to activate PI3K or Rac1, respectively. During the 740Y-P treatment, the p-PI3K levels were upregulated (Fig. 6A and G), resulting in the elevation of the downstream target, Rac1-GTP (Fig. 6A and H). It is worth mentioning that p-PI3K upregulation by 740Y-P did not affect NET expression (Fig. S5C-D), elucidating a direct interaction between PI3K and Rac1. Furthermore, treatment with 740Y-P or ML-099 led to the activation of the macrophages PI3K/Rac1 signaling pathway (Fig. 6A and H), which counteracted the inhibited effects of NETs in diabetic mouse wounds, restoring impaired efferocytosis, and thus reducing the enlarged area of ACs to levels comparable to those in normal mice (Fig. 6I–K). Concurrently, activation of the PI3K/Rac1 pathway by administration with 740Y-P led to rapid wound healing in diabetic mice (Fig. S5E-F). Altogether, these results suggested that NETs in diabetic mouse wounds affect efferocytosis via PI3K/Rac1 signaling, resulting in delayed wound healing.

**Fig. 7:**
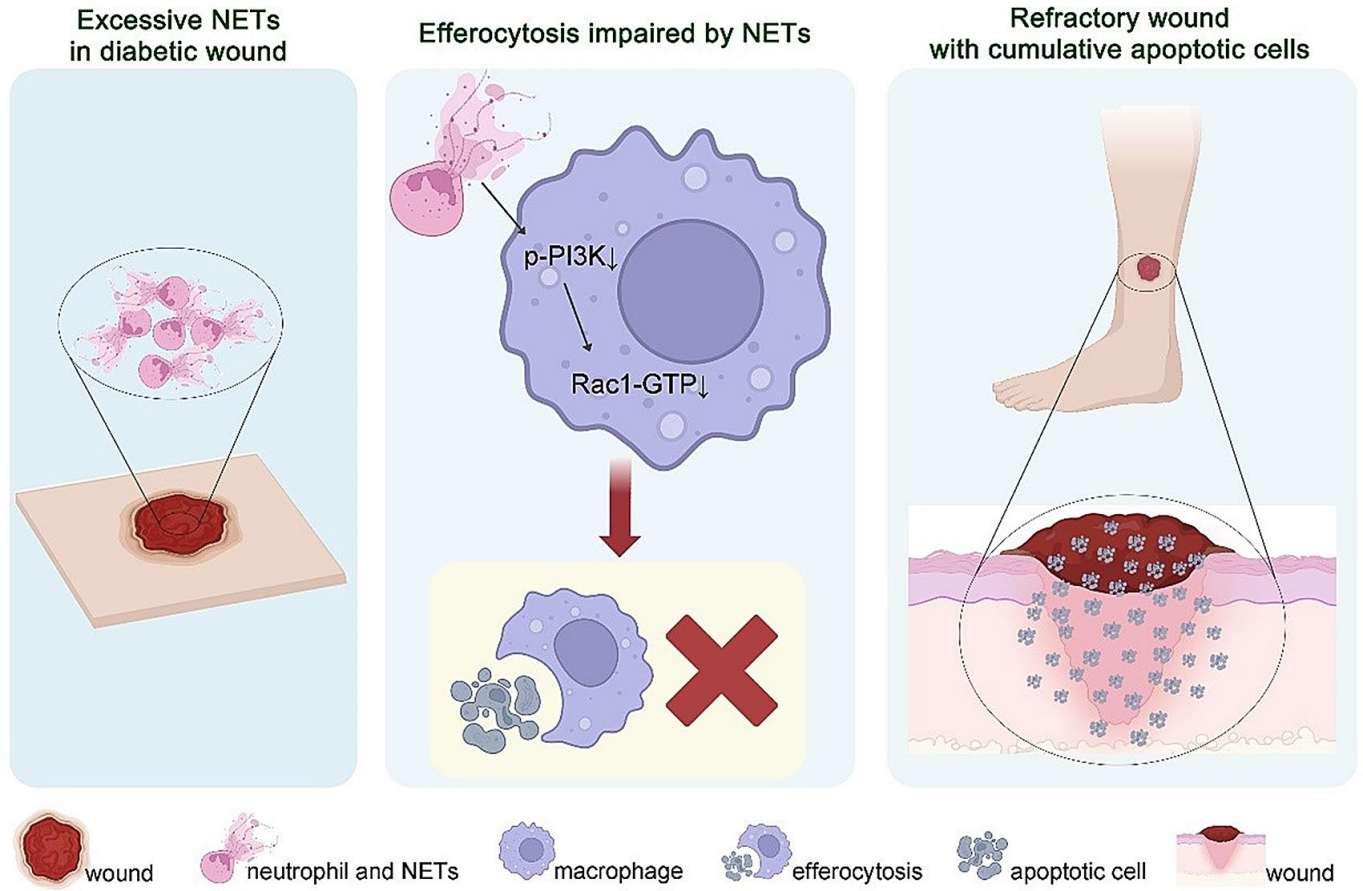
Mechanism diagram showing the interrelationships between NETs, the PI3K/Rac1 signaling pathway and macrophage efferocytosis in diabetic wound.

## Discussion

This study investigated the hypothesis that NETs critically mediate efferocytosis of ACs in diabetic wounds (Fig. 7). The findings indicated that elevated NET levels in diabetic wounds mediate macrophage efferocytosis through the PI3K/Rac1 pathway. This interaction highlights the complex cellular crosstalk between neutrophils and macrophages, where neutrophil-derived NETs can act as a double-edged sword, promoting pathogen clearance while simultaneously impairing macrophage function. Inhibition of NET formation or enhancement of the PI3K/Rac1 signaling pathway can ameliorate the impaired efferocytosis and reduce the enlarged ACs area in diabetic mouse wounds, emphasizing the potential of targeting NETs and macrophage signaling as therapeutic strategies to improve wound resolution in diabetes.

Macrophages are essential in the inflammatory milieu by facilitating the clearance of ACs by efferocytosis, a specialized phagocytic process; the Greek meaning of this term is “to carry to the grave” [23]. Efferocytosis by macrophages is instrumental in the resolution of inflammation by engaging specific receptors and activating downstream signaling pathways. This is achieved by (i) engulfing ACs, thereby preventing their lysis and the subsequent release of inflammatory mediators, and (ii) secreting anti-inflammatory cytokines such as interleukin-10 and transforming growth factor-beta, which dampen pro-inflammatory responses [23]. The efficacy of efferocytosis can be influenced by various signals within the inflammatory milieu, including those from dying cells, which act as direct inhibitors or enhancers [24–27]. Notably, persistent inflammatory signaling in macrophages leads to impaired clearance of ACs, which is associated with the progression of autoimmune and chronic inflammatory conditions such as systemic lupus erythematosus [28, 29], type I diabetes [30, 31], chronic obstructive pulmonary disease [32–34], and cardiovascular diseases [35, 36]. However, the precise mechanisms underlying efferocytosis in diabetic wounds are not fully understood. The hyperglycemic milieu and associated metabolic perturbations characteristic of diabetes may potentially alter macrophage function and efferocytosis capacity, thereby contributing to the chronic nature of wounds in these patients. It is worthwhile to clarify the role of efferocytosis in diabetic wound healing and to determine if targeted manipulation of macrophage efferocytosis can present a novel therapeutic strategy.

Diabetes has been demonstrated to significantly impair wound healing, thereby increasing morbidity and mortality rates [1, 4, 6]. In the early stages of wound healing, neutrophils are the predominant leukocytes exerting antimicrobial effects by generating NETs; however, this process also induces tissue damage [7, 8, 11]. Both diabetic patients and mouse diabetic models exhibit an increased propensity for neutrophil-mediated NETosis, resulting in delayed wound healing [9]. Furthermore, studies have identified that macrophage efferocytosis is diminished in diabetic wounds, leading to a substantial accumulation of uncleared ACs, which consequently results in chronic inflammation [4–6]. The wounds of STZ-induced diabetic mice in this study exhibited elevated levels of citrullinated histone H3 and reduced efferocytosis, which were ameliorated following Cl-Ad administration. Moreover, in vitro efferocytosis assays demonstrated that NETs impair the efferocytosis capacity of macrophages. This study firstly provided the evidence that NETs within STZ-induced diabetic mouse wounds impair macrophage efferocytosis. Furthermore, it was found that these macrophages retained impaired efferocytosis capacity even after removal from the diabetic microenvironment through in vitro efferocytosis assays and in vivo adoptive transfer, where macrophages isolated from diabetic wound environments were transplanted into normal mouse wounds. These indicated that the impact of NETs on macrophage efferocytosis in diabetic wounds is persistent.

The uptake of dead cells is mediated by the Rho family of small GTPases, including RhoA, Rac, and Cdc42, which oscillate between inactive GDP-bound and active GTP-bound states under the influence of specific guanine nucleotide exchange factors. RhoA activation can inhibit the phagocytosis of ACs, whereas Rac and Cdc42 facilitate this process [21]. Upon identification of dead cells by phagocytic cells, the Rho family relocates to the plasma membrane, initiating a cascade of signaling pathways. Subsequently, the cytoskeleton undergoes reorganization through a dynamic network of actin filaments beneath the plasma membrane, resulting in membrane invagination, localized membrane protrusion, the formation of a phagocytic cup on the side facing the ACs, and ultimately culminating in phagosome formation [21, 37]. This process is primarily regulated by two key complexes: CrkII/ELMO/Dock180 and ABCA1/GULP, both of which ultimately activate Rac1, initiating cytoskeletal rearrangement and subsequent phagocytosis [21]. The findings of this study underscored the critical role of Rac1 in the phagocytic process in the context of diabetic wounds, suggesting that NETs are likely to influence Rac1 activation levels. We hypothesized that NETs present in diabetic wounds may emit inhibitory signals that regulate Rac1 activation of macrophages, thus suppressing efferocytosis. Then, the in vitro and in vivo experimental results of this study demonstrated that Rac1 activation levels decreased in the presence of NETs, consequently inhibiting macrophage efferocytosis, which preliminarily supports our hypothesis.

Activation of the PI3K pathway via its product 3′PI is crucial for the phagocytosis of large particles by cells [22]. Several disease studies, including in vitro models of COPD/emphysema [38], acute respiratory distress syndrome (ARDS) [39], a mouse carotid plaque model [40], a mouse hypertension-induced heart failure model due to cardiac remodeling [41], and a mouse gout model [42], have suggested that reduced PI3K activation correlates with decreased macrophage efferocytosis. A study by Huan Tao et al. reported that the absence of macrophage SR-BI in a mouse arterial plaque model promotes defective efferocytosis signaling via the Src/PI3K/Rac1 pathway, leading to increased plaque size, necrosis, and inflammation [20]. Studies have revealed that macrophages in diabetic mouse wounds exhibit low levels of PI3K/Akt signaling, which is associated with altered polarization [43, 44]. Based on current evidence, it is hypothesized that NETs in STZ-induced diabetic mice wounds inhibit the activation of the PI3K/Rac1 signaling pathway in macrophages, thereby diminishing efferocytosis. This study reported that NETs affect the activation of signaling molecules in macrophages, such as PI3K and Rac1, which is critical for macrophage efferocytosis in the clearance of ACs from wound sites through a series of in vitro and in vivo validation experiments. Inhibiting NET formation or activating the PI3K/Rac1 pathway in diabetic mice wounds can enhance efferocytosis and reduce AC retention, which is associated with the resolution of inflammation and effective wound remodeling. Notably, in vitro efferocytosis assays revealed that simultaneous activation of PI3K and inhibition of Rac1 in the presence of NETs did not enhance efferocytosis, suggesting that Rac1 may be the sole downstream pathway through which PI3K mediates the reduction of macrophage efferocytosis by NETs.

While our study highlights the role of NETs and the PI3K/Rac1 pathway in macrophage efferocytosis during diabetic wound healing, several limitations should be noted. Firstly, our STZ-induced type 1 diabetes model does not fully replicate the complexity of clinical diabetic wounds, which often involve type 2 diabetes and chronic inflammation. Future studies should explore these mechanisms in models that better mimic the clinical setting. Secondly, wound healing is a dynamic process involving multiple cell types, including fibroblasts, keratinocytes, and endothelial cells. Although our study focused on macrophages, the effects of NETs and PI3K/Rac1 modulation on other cell types remain unclear. Future research should investigate these interactions to provide a more comprehensive understanding. Additionally, the non-specific effects of inhibitors and activators on other signaling pathways warrant further validation through alternative approaches. Our study also lacks in-depth mechanistic research, such as the identification of specific interaction proteins and regulatory networks involved in NETs-mediated macrophage efferocytosis. Further investigation will help translate our findings into effective therapeutic strategies for diabetic wound healing.

## Conclusion

In summary, this study firstly provided the evidence that excessive NETs in STZ-induced diabetic mouse wounds lead to sustained impairment of macrophage efferocytosis, resulting in the accumulation of ACs, which is detrimental to the resolution of inflammation and normal wound healing. Furthermore, NETs inhibit efferocytosis signaling through the regulation of the macrophage PI3K/Rac1 pathway. Both the suppression of NET formation and the activation of the PI3K/Rac1 pathway can restore macrophage efferocytosis. The accumulation of ACs resulting from diminished NETs-mediated efferocytosis may represent a critical factor in the progression of diabetic wounds into chronic ulcers. Consequently, these findings imply that interventions aimed at modulating the excessive formation of NETs or their participation in the PI3K/Rac1 pathway may serve as potential therapeutic targets. Additionally, these findings offer preliminary evidence and a novel research direction for further investigation of the inflammatory phase development in diabetic wounds.

## Electronic supplementary material

Below is the link to the electronic supplementary material.

Supplementary Material 1

Supplementary Material 2

## Data Availability

No datasets were generated or analysed during the current study.

## Declarations

### Authors’ relationships and activities

The authors declare that there are no relationships or activities that might bias, or be perceived to bias, their work.

## Abbreviations

3′PI: 3′ phosphoinositides
ACs: Apoptotic cells
BCA: Bicinchoninic acid
DMEM: Dulbecco’s Modified Eagle Medium
ELISA: Enzyme-linked immunosorbent assay
FBS: Fetal bovine serum
FcR: Fcγ receptors
H3Cit: Citrullinated histone H3
NETs: Neutrophil extracellular traps
PAD: Peptidylarginine deiminase
PI3K: Phosphoinositide 3-kinase
Rac1: Ras-related C3 botulinum toxin substrate 1
SLE: Systemic lupus erythematosus
STZ: Streptozotocin
TGF-β: Transforming growth factor-beta
