# Supplementary material for: The efferocytosis dilemma: how neutrophil extracellular traps and PI3K/Rac1 complicate diabetic wound healing
Source: Cell Commun Signal. 2025 Feb 21;23:103. doi: 10.1186/s12964-025-02092-4 (PMC11844175; doi:10.1186/s12964-025-02092-4)

Figure. 3 A. β-actin

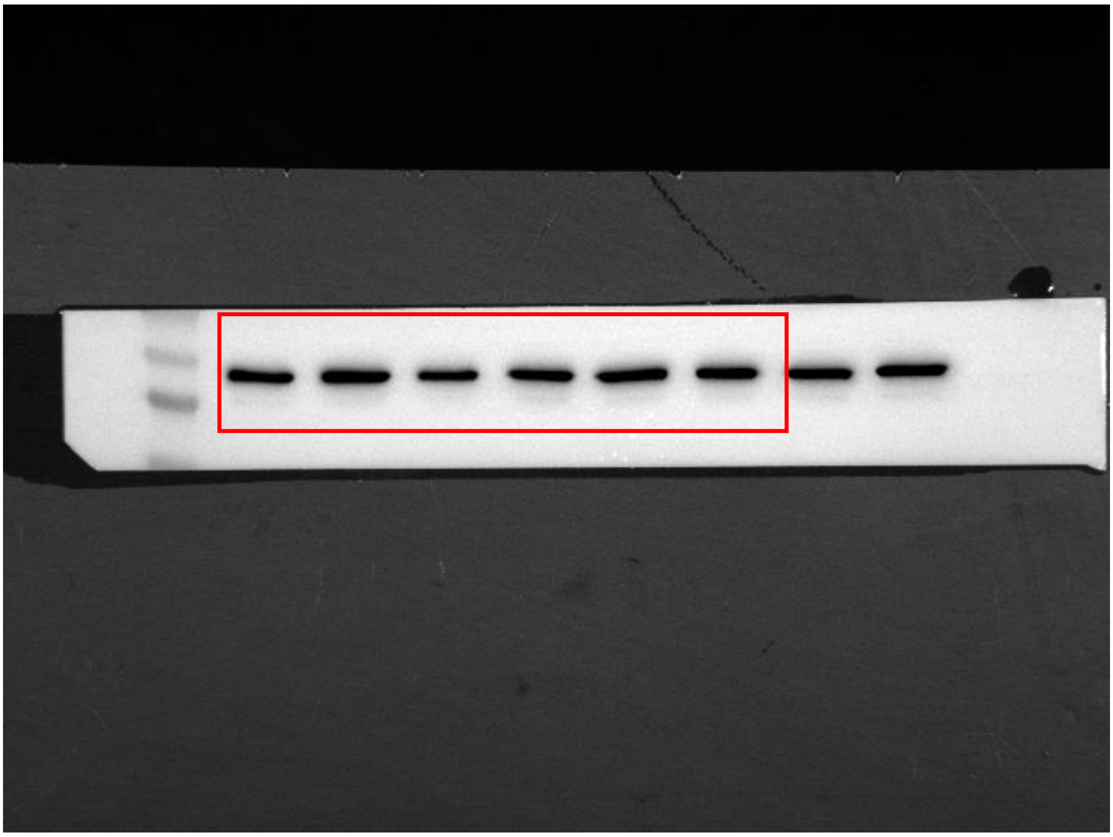

Figure. 3 A. total Rac1

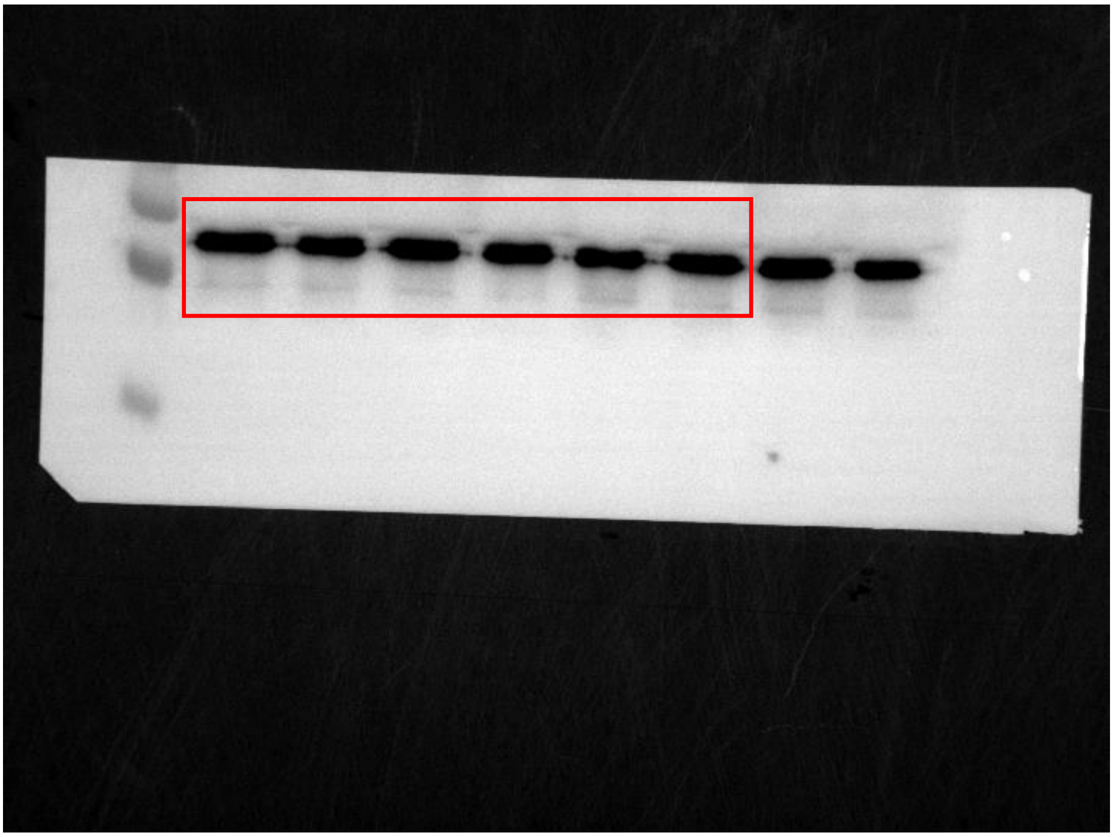

Figure. 3 A. Rac1-GTP

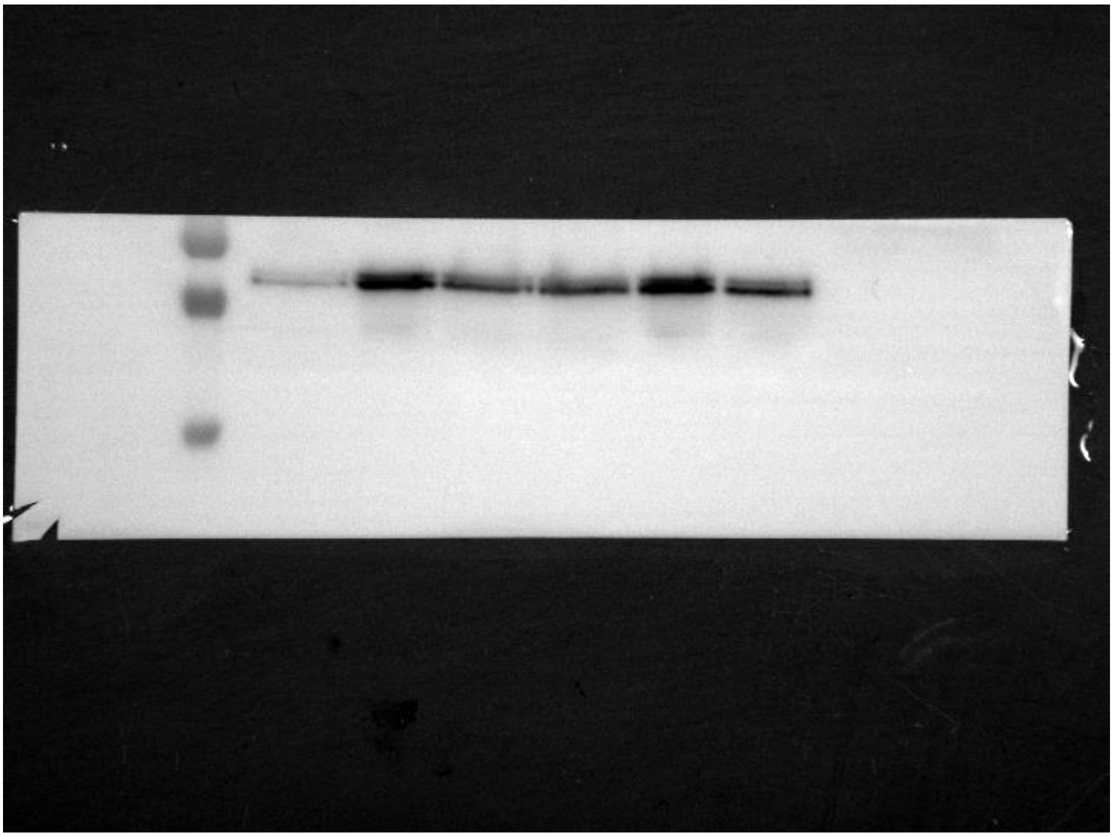

Figure. 4 A. β-actin

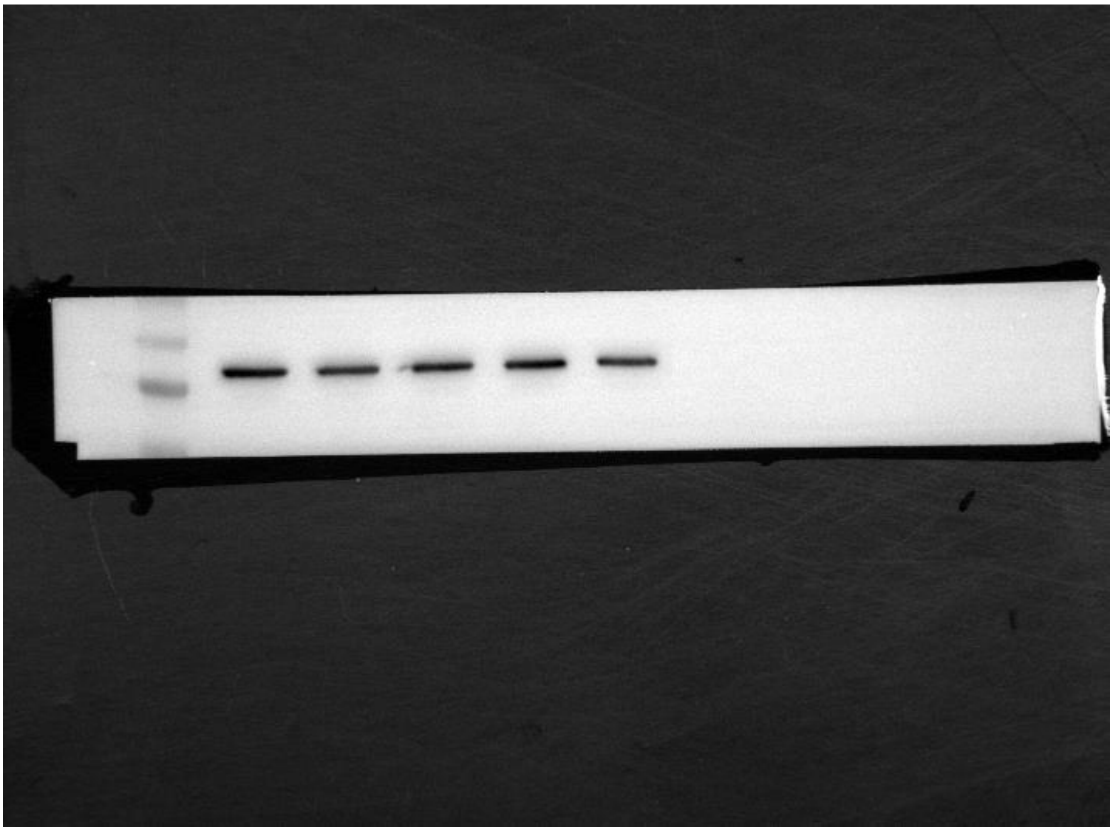

Figure. 4 A. total Rac1

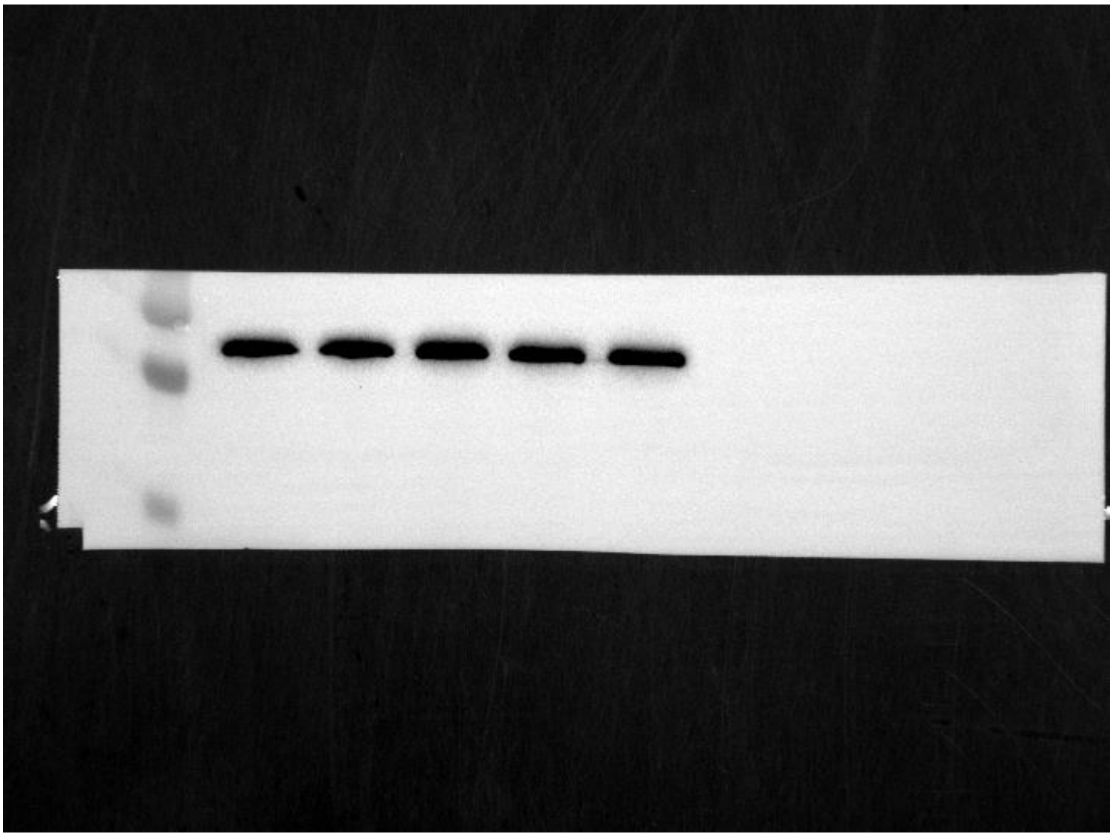

Figure. 4 A. Rac1-GTP

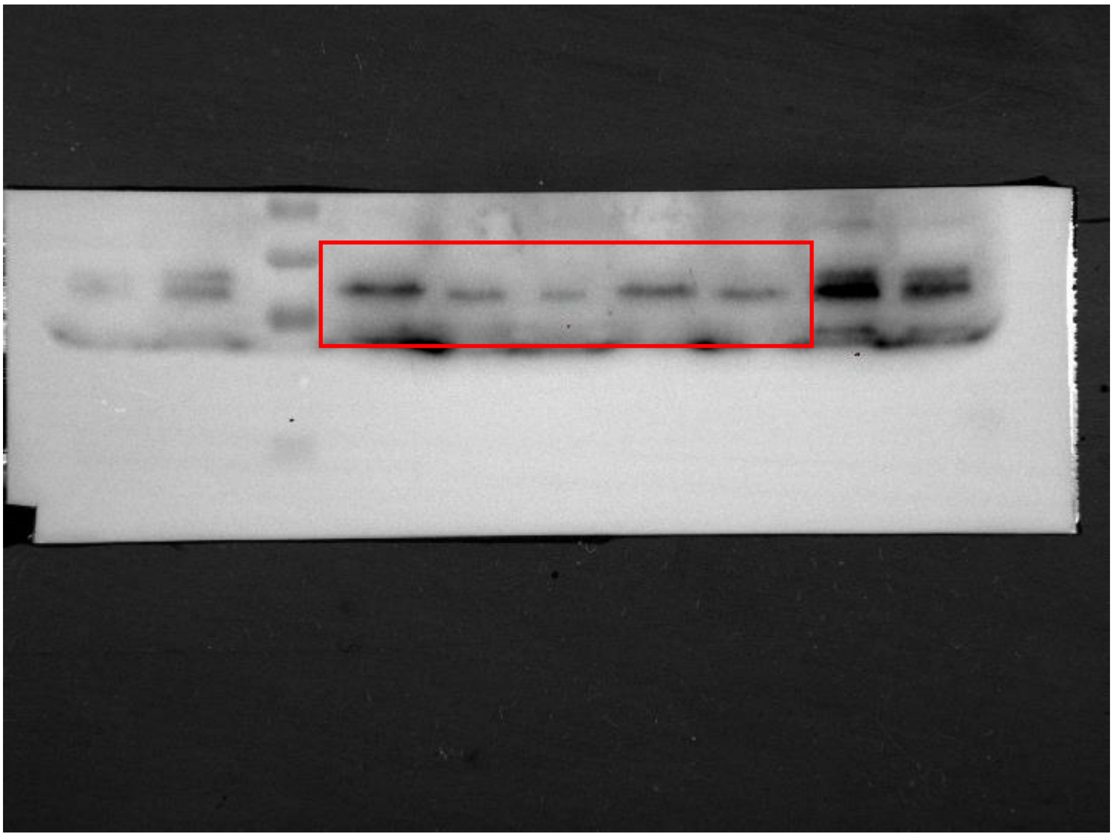

Figure. 5 A. p-PI3K

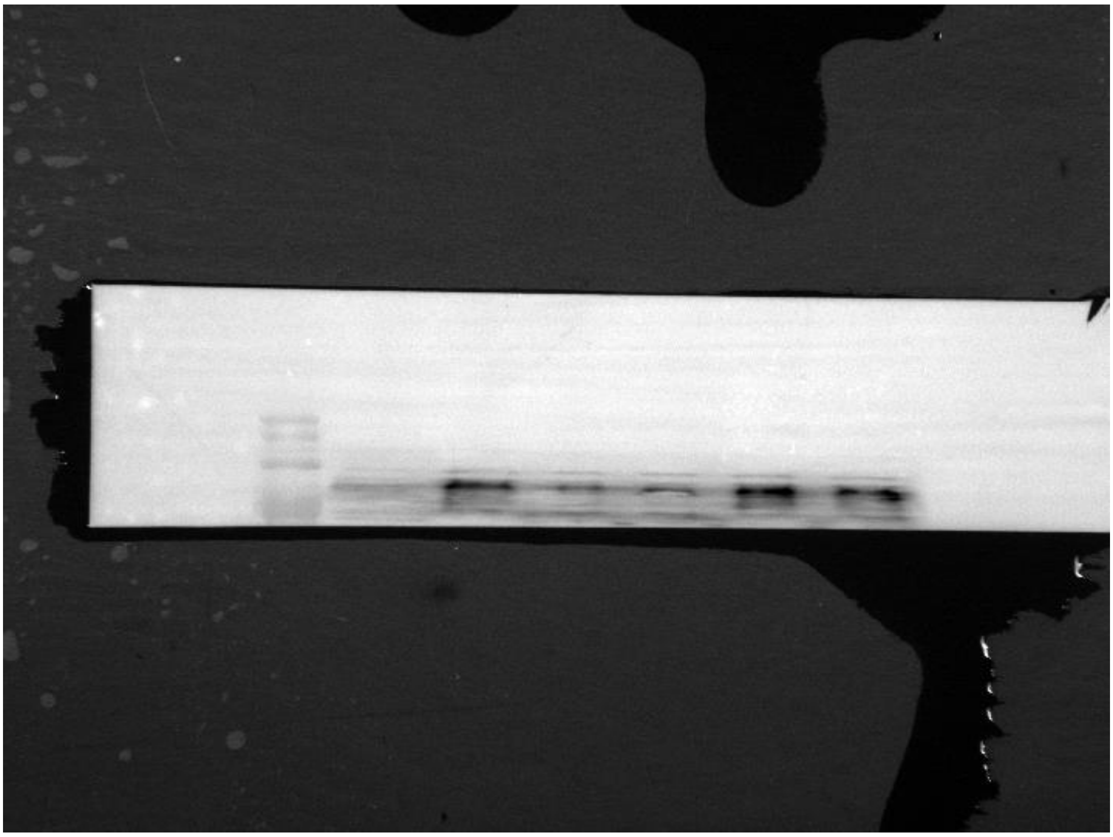

Figure. 5 A. β-actin

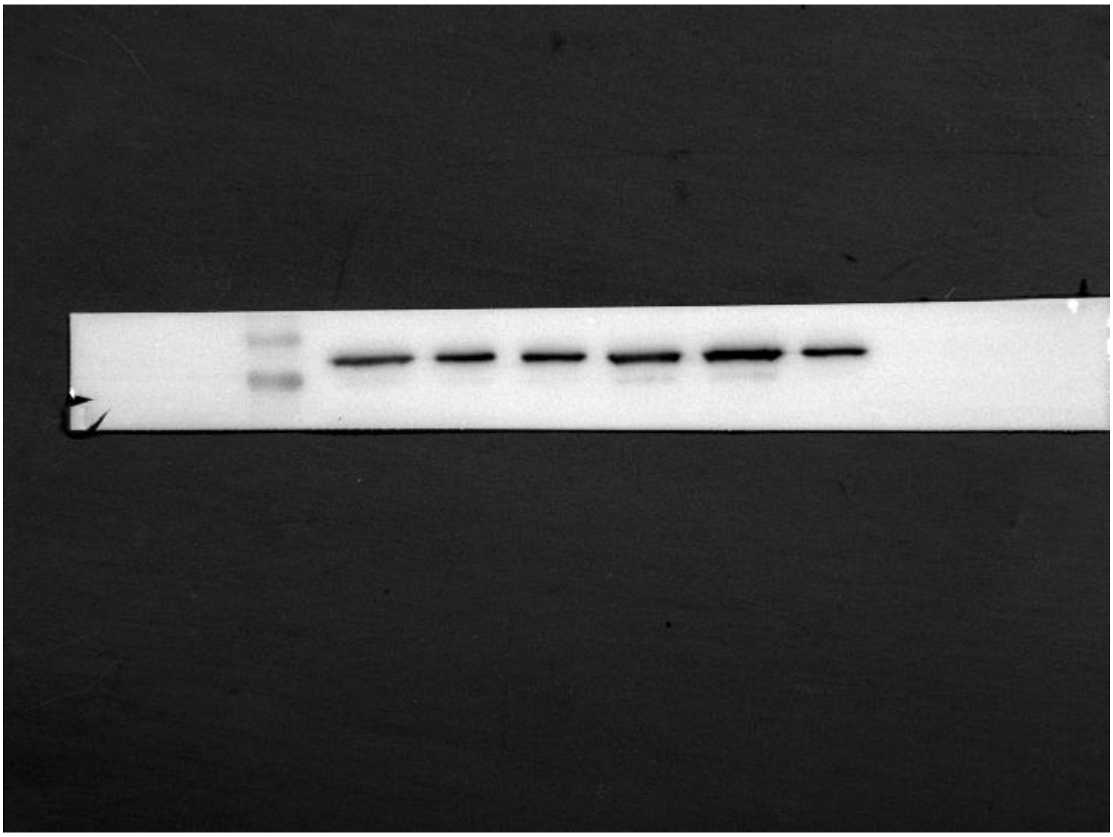

Figure. 5 A. total Rac1

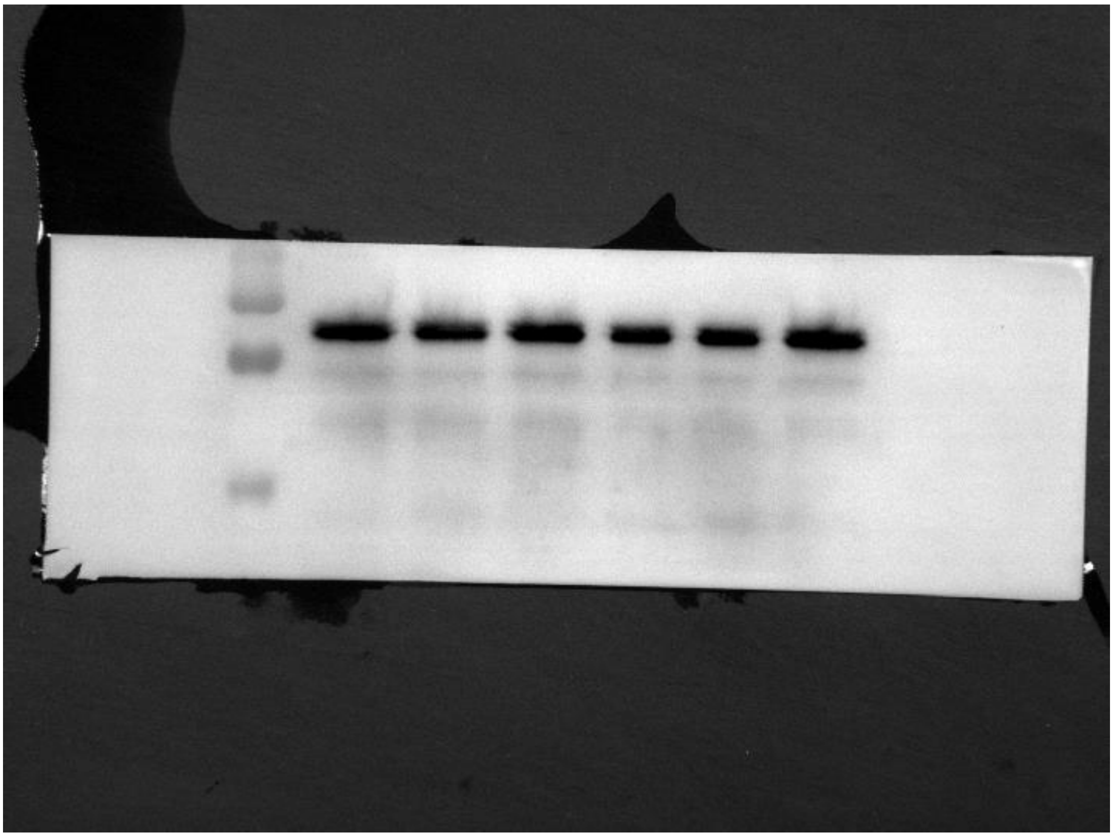

Figure. 5 A. Rac1-GTP

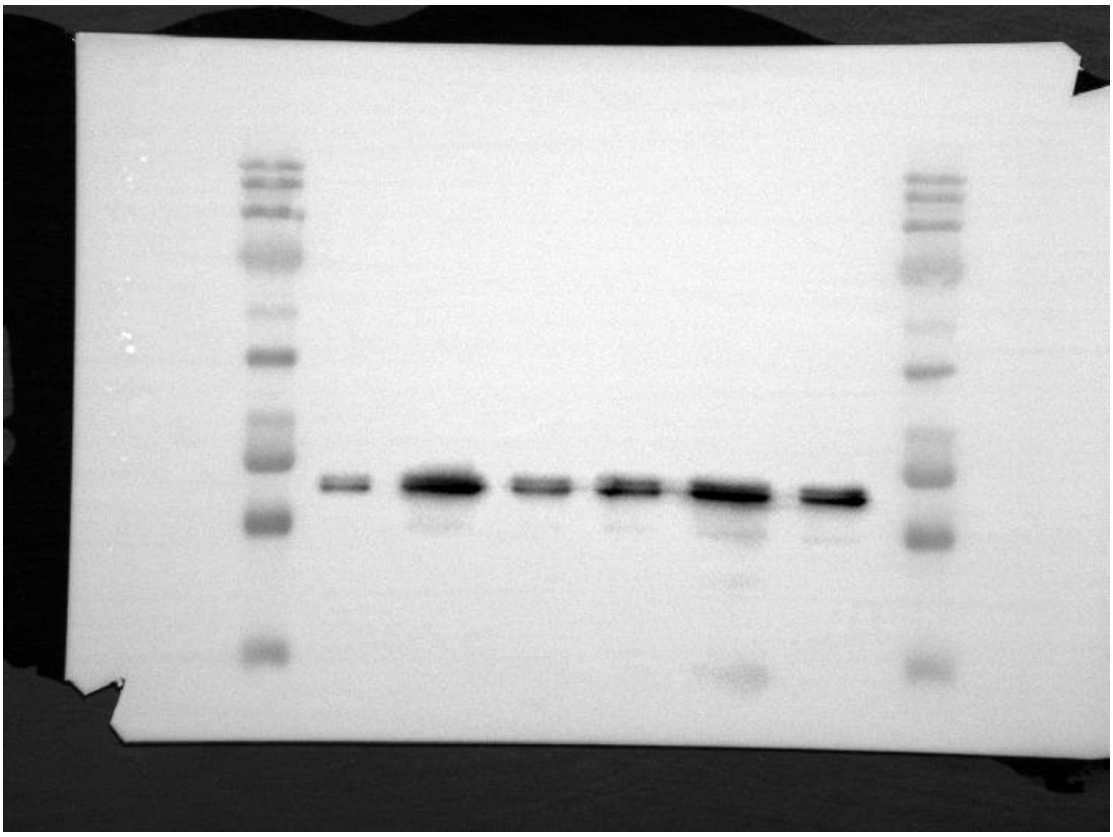

Figure. 6 A. p-PI3K

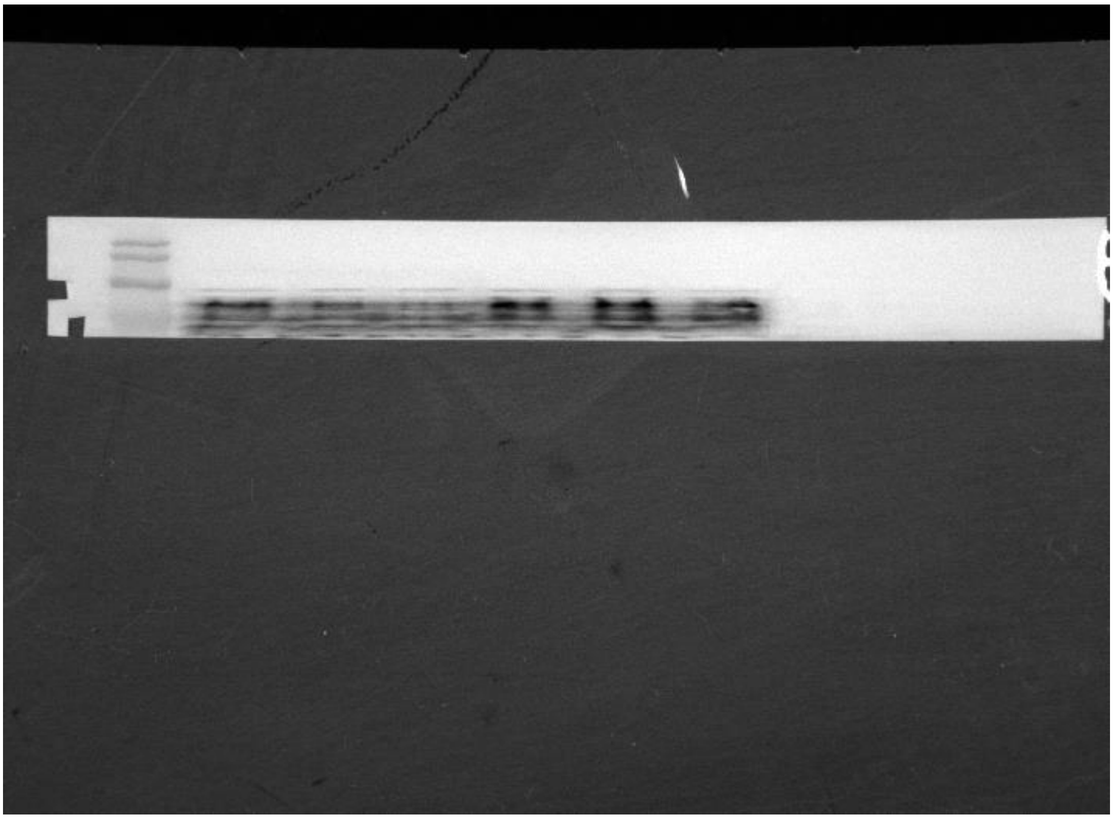

Figure. 6 A. β-actin

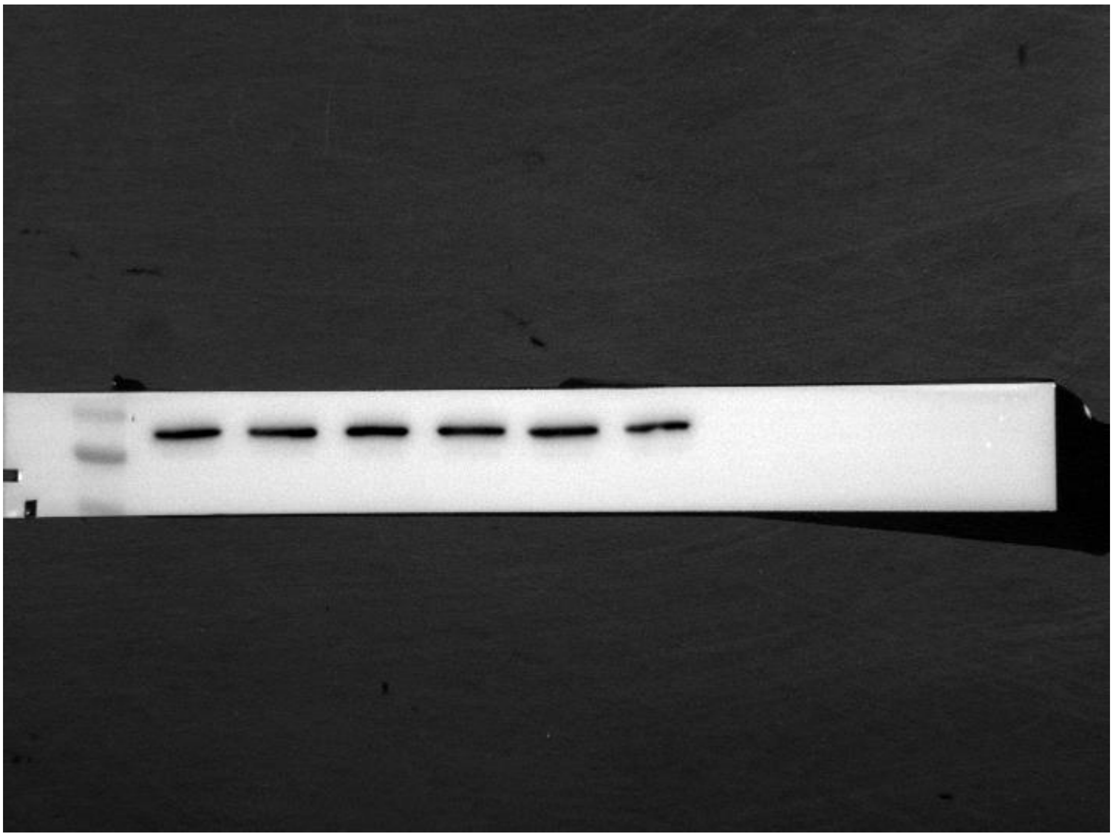

Figure. 6 A. total Rac1

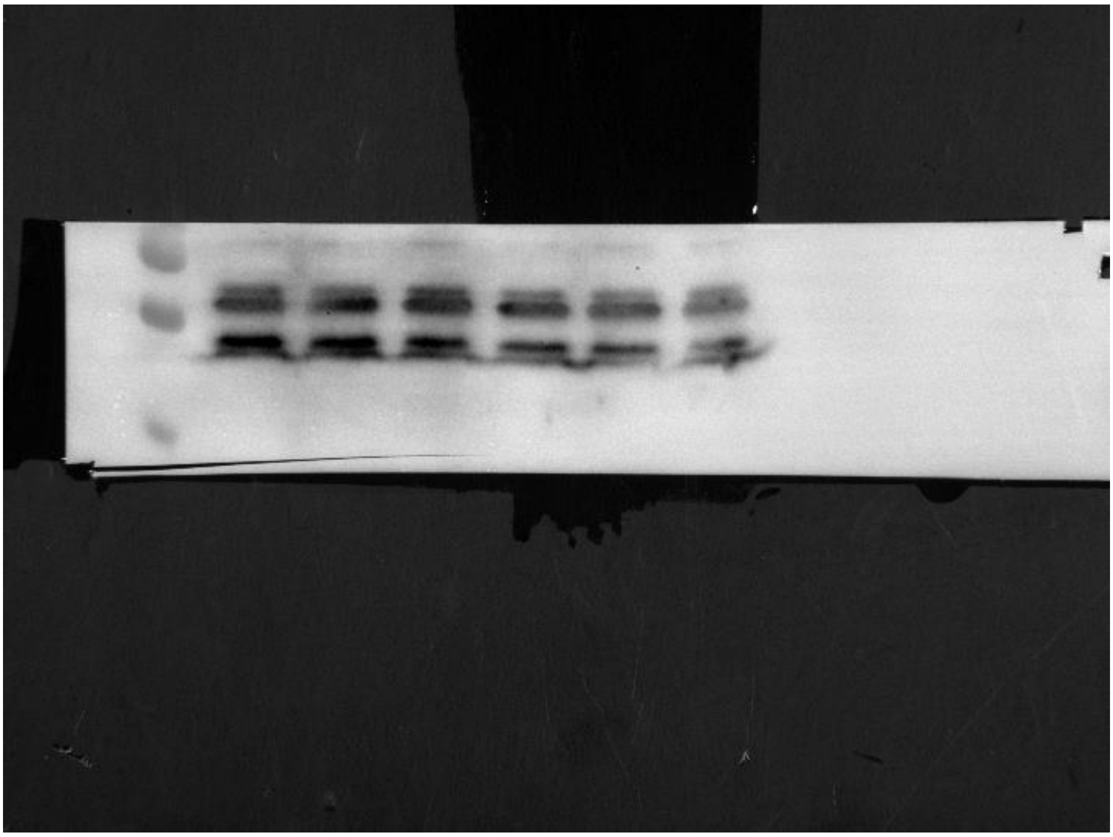

Figure. 6 A. Rac1-GTP

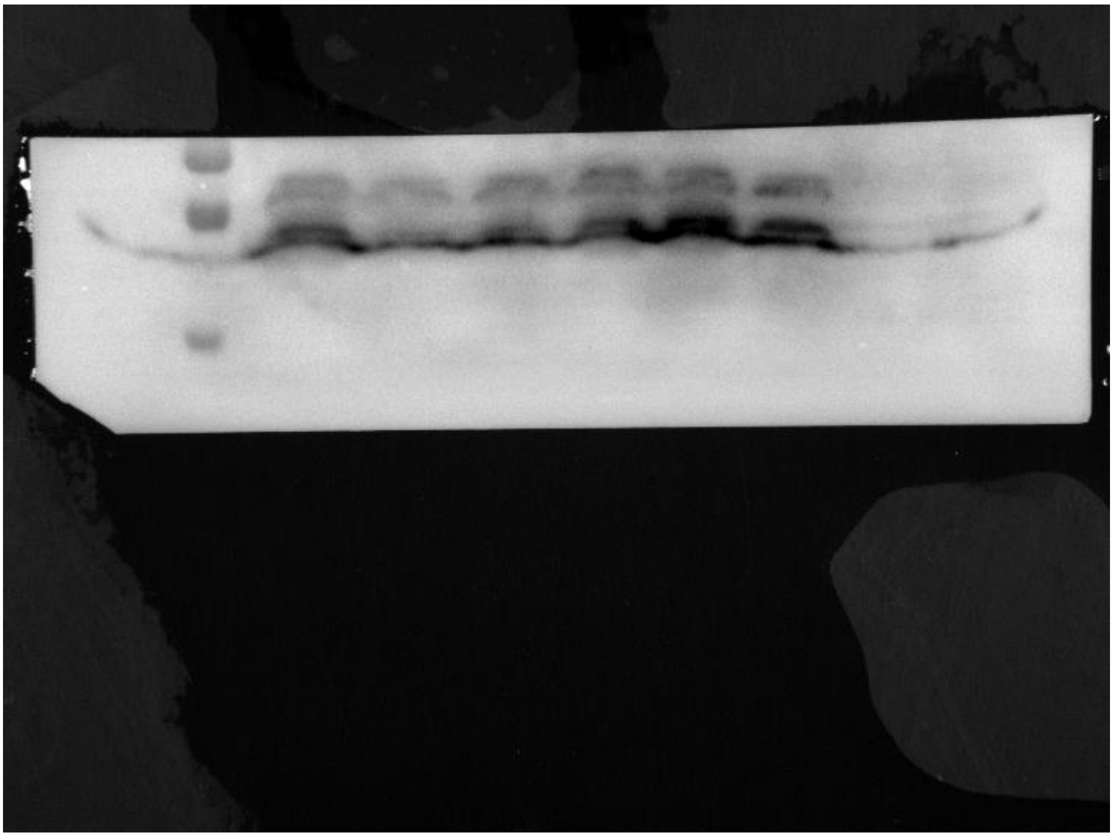

Supplement: Supplementary file 1 — Supplementary Material 1 [file 12964_2025_2092_MOESM1_ESM.pdf]
