# Supplementary material for: The efferocytosis dilemma: how neutrophil extracellular traps and PI3K/Rac1 complicate diabetic wound healing
Source: Cell Commun Signal. 2025 Feb 21;23:103. doi: 10.1186/s12964-025-02092-4 (PMC11844175; doi:10.1186/s12964-025-02092-4)
Supplement: Supplementary file 2 — Supplementary Material 2 [file 12964_2025_2092_MOESM2_ESM.docx]

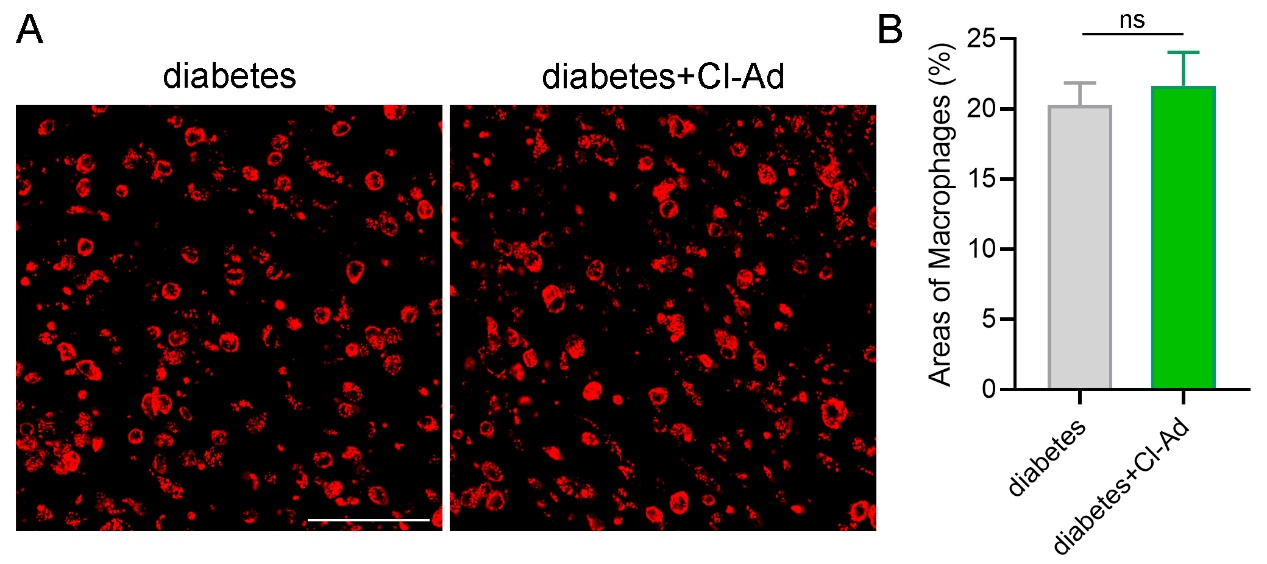


Fig. S1 Supplementary images for effect on recruitment of macrophages by Cl-Ad.

(A) Representative immunofluorescence images of macrophages infiltration in the wounds of diabetic mice and diabetic mice treated with Cl-Ad. n=5 per group. Scale bar, 50 μm. (B) F4/80+ was defined as macrophage. This is a graph of areas of macrophages quantitative evaluation in (A). Data were represented as mean ± SEM; ns indicated no significant difference; *P < 0.05; **P < 0.01; ***P < 0.001.


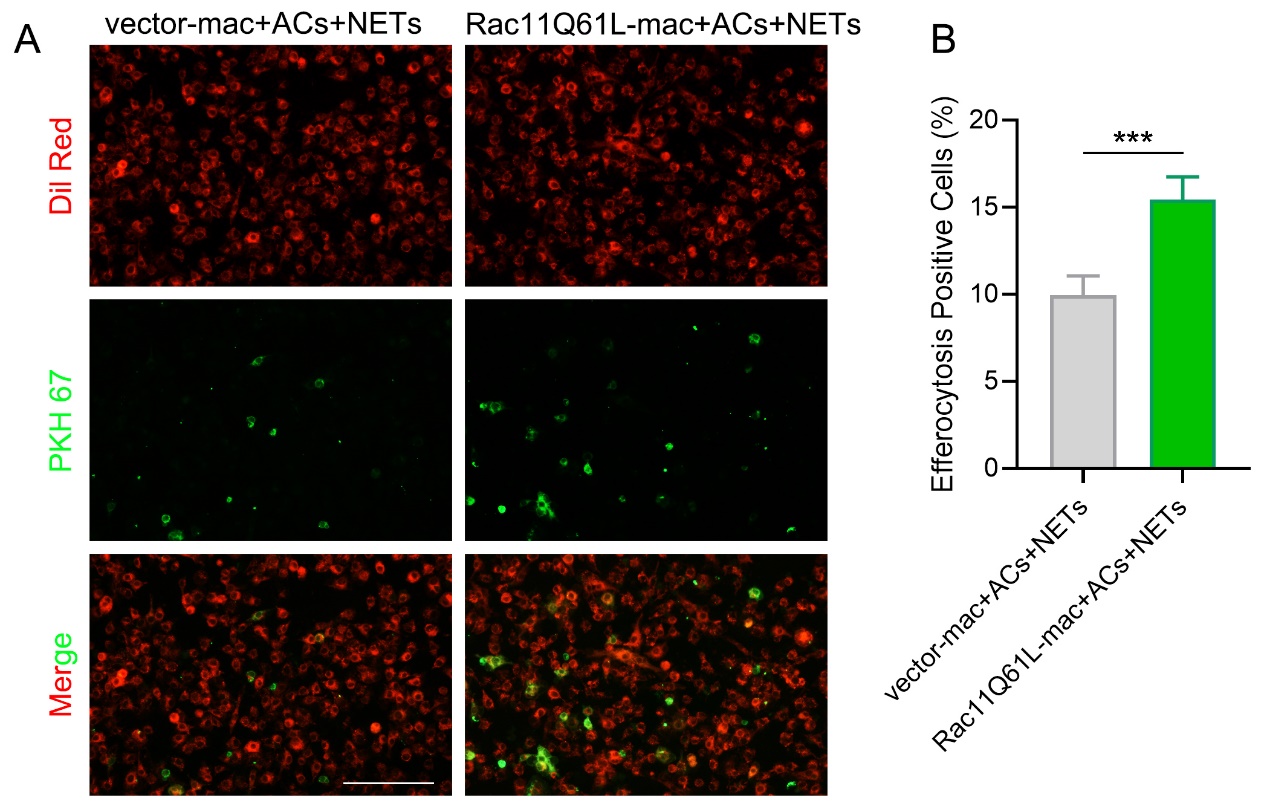


Fig. S2 Supplement for examination that role of Rac1-GTP in efferocytosis *in vitro.*

(A) Fluorescent images demonstrating Raw264.7 cells undergoing efferocytosis by overexpressing Rac11Q61L or control vector. Scale bar, 50 µm. (B) Quantitative evaluation of the findings in (A).


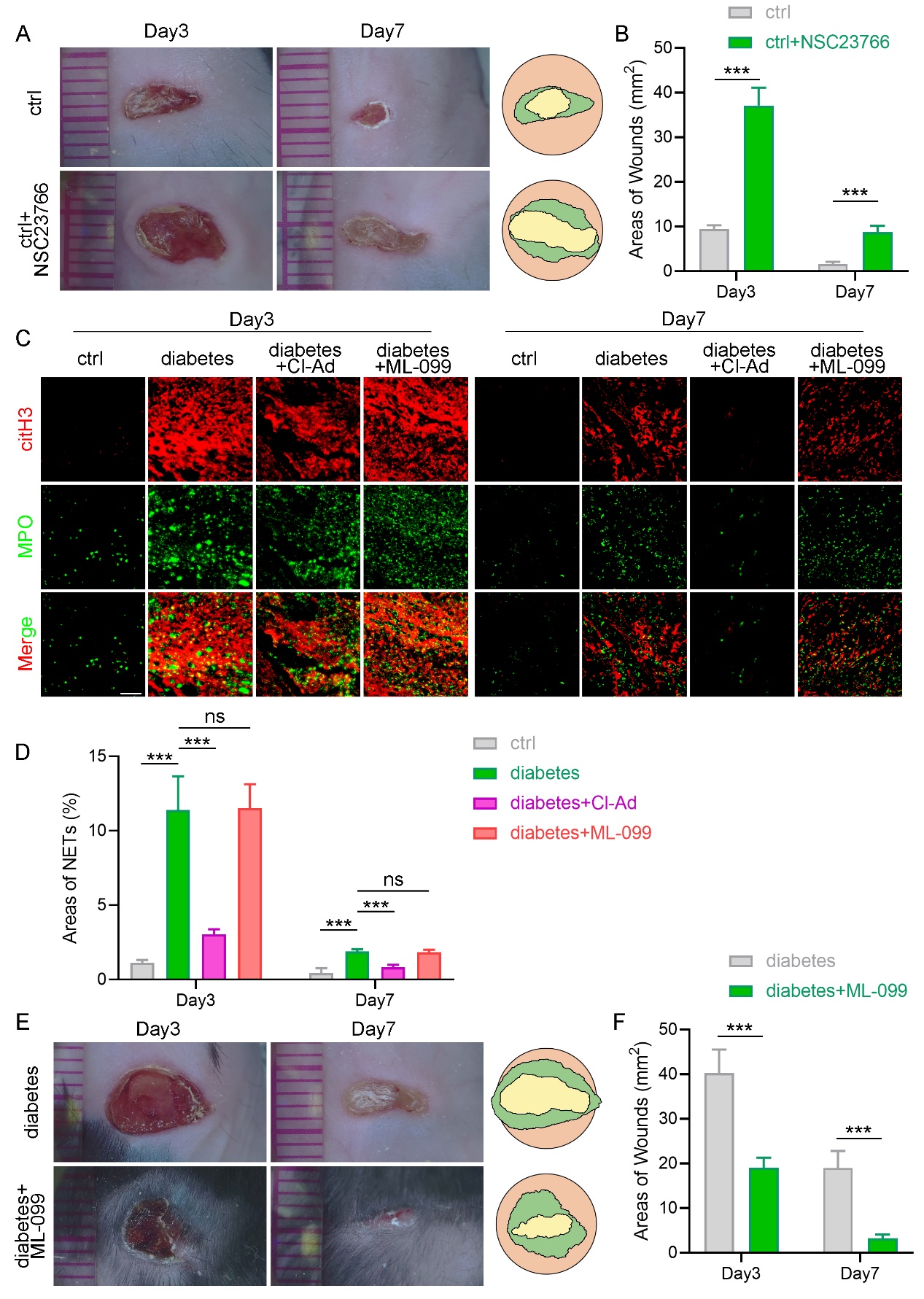


Fig. S3 Supplement for examination that exploring role of NETs and Rac1-GTP in efferocytosis *in vivo.*

(A) The gross view of wounds in the normal mice and NSC23766-treated normal mice were observed on days 3 and 7. (B) Measuring the extent of wound healing in (A). (C) Representative immunofluorescence images of NETs formation in the wounds of normal mice, diabetic mice and diabetic mice treated with Cl-Ad, ML-099. n=5 per group. Scale bar, 25 μm. (D) Quantitative evaluation of the findings in (C). (E) The gross view of wounds in the diabetic mice and ML-099 treated diabetic mice were observed on days 3 and 7. (F) Measuring the extent of wound healing in (E). Data were represented as mean ± SEM; ns indicated no significant difference; *P < 0.05; **P < 0.01; ***P < 0.001.


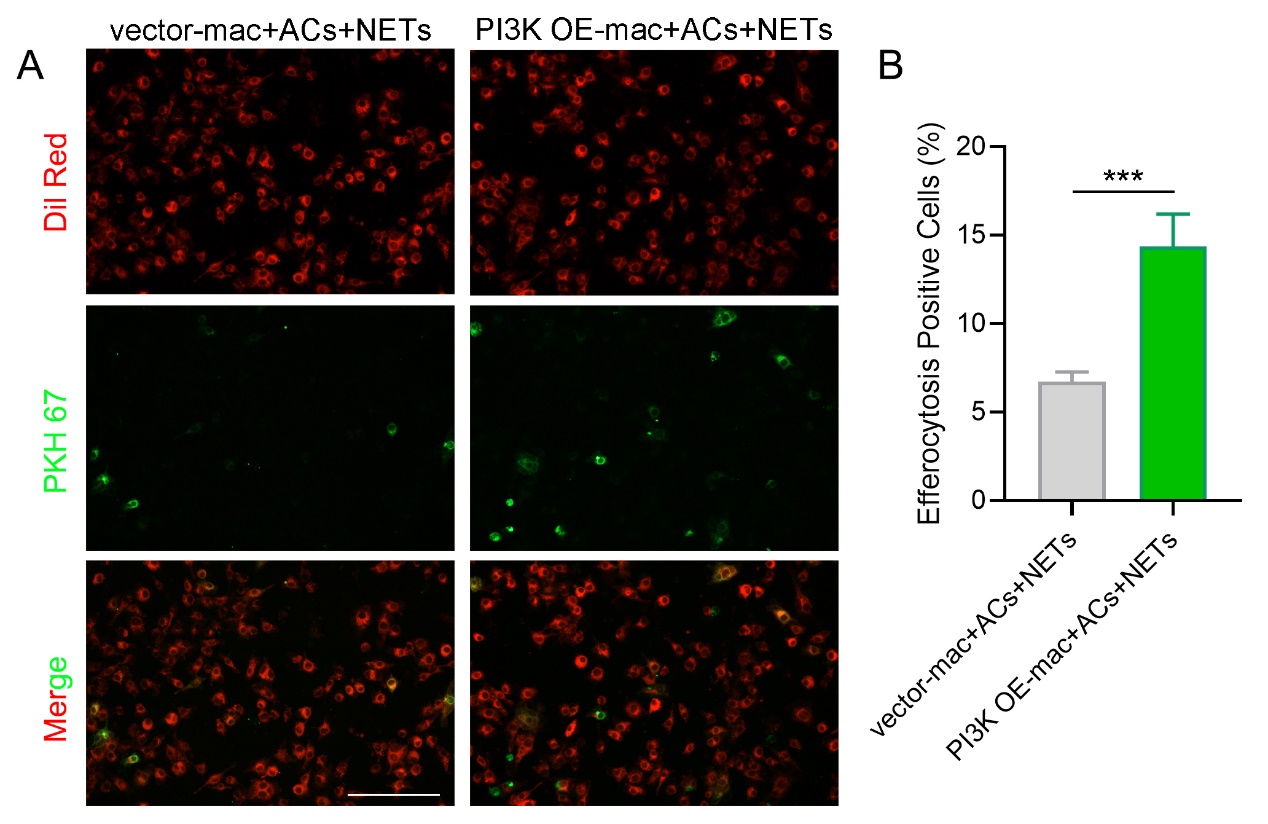


Fig. S4 Supplement for examination that role of p-PI3K in efferocytosis *in vitro.*

(A) Fluorescent images demonstrating Raw264.7 cells undergoing efferocytosis by overexpressing PI3K or control vector. Scale bar, 50 µm. (B) Quantitative evaluation of the findings in (A).


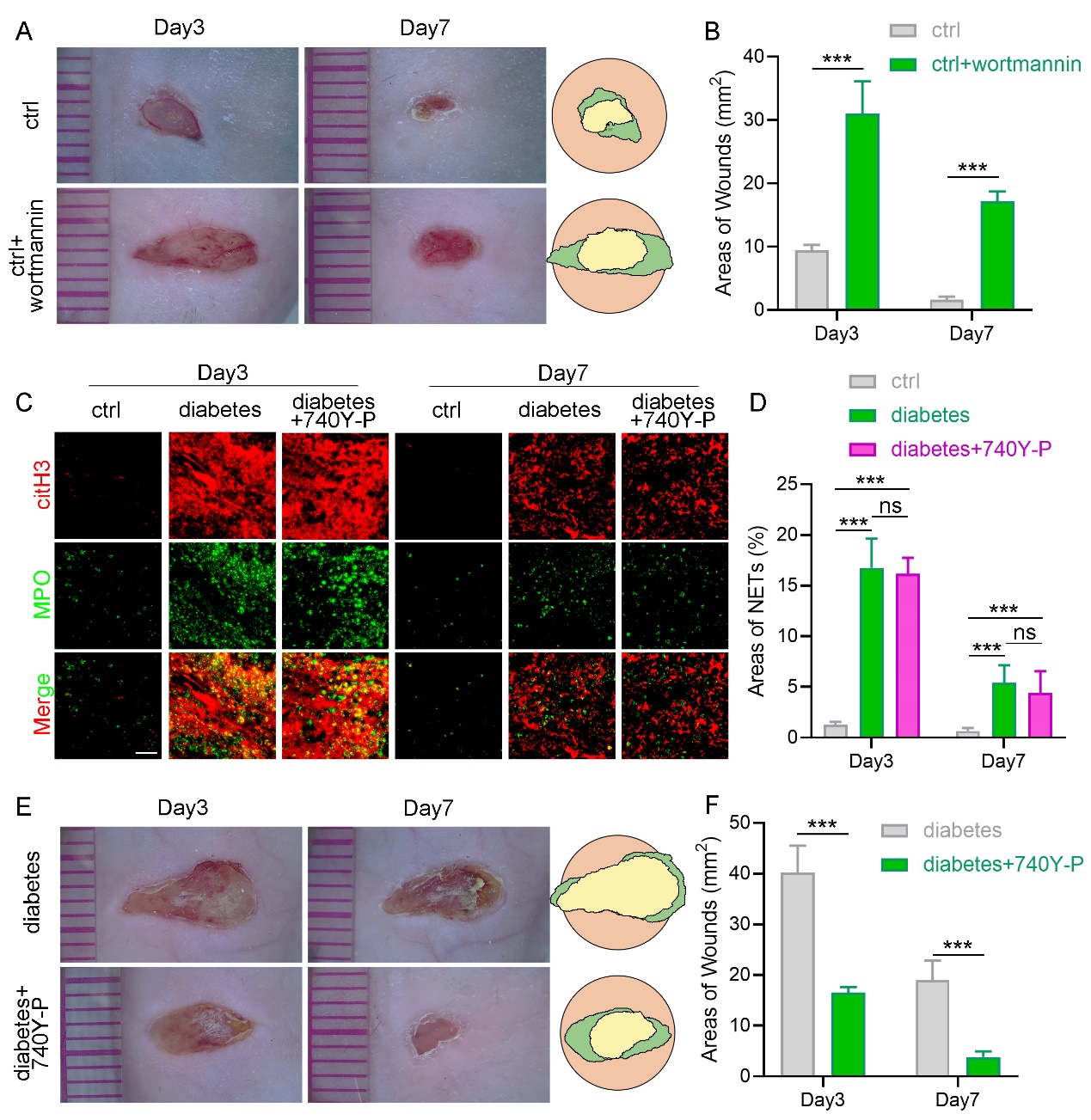


Fig. S5 Supplement for examination that exploring role of NETs, p-PI3K and Rac1-GTP in efferocytosis *in vivo.*

(A) The gross view of wounds in the normal mice and wortmannin-treated normal mice were observed on days 3 and 7. (B) Measuring the extent of wound healing in (A). (C) Representative immunofluorescence images of NETs formation in wounds of normal mice, diabetic mice and diabetic mice treated with740Y-P. n=5 per group. Scale bar, 25 μm. (D) Quantitative evaluation of the findings in (C). (E) The gross view of wounds in the diabetic mice and 740Y-P treated diabetic mice were observed on days 3 and 7. (F) Measuring the extent of wound healing in (E). Data were represented as mean ± SEM; ns indicated no significant difference; *P < 0.05; **P < 0.01; ***P < 0.001.
